# Substance Use During Imprisonment in Low- and Middle-Income Countries

**DOI:** 10.1093/epirev/mxx016

**Published:** 2018-03-23

**Authors:** Adrian P Mundt, Gergő Baranyi, Caroline Gabrysch, Seena Fazel

**Affiliations:** 1 Medical Faculty, Universidad Diego Portales, Santiago, Chile; 2 Medical School, Universidad San Sebastián, Puerto Montt, Chile; 3 Center for Research on Environment Society and Health, School of Geosciences, University of Edinburgh, Edinburgh, United Kingdom; 4 Institute and Polyclinic for Occupational and Social Medicine, Technische Universität Dresden, Germany; 5 Department of Psychiatry and Psychotherapy Campus Mitte, Charité Universitätsmedizin Berlin, Berlin, Germany; 6 Department of Psychiatry, Oxford University, Oxford, United Kingdom

**Keywords:** alcohol, illicit drugs, injection drug use, nicotine, prevalence, prison, substance use

## Abstract

Substance use disorders are among the most common health problems of people involved with the criminal justice system. Scaling up addiction services in prisons is a global public health and human rights challenge, especially in poorly resourced countries. We systematically reviewed the prevalence of substance use in prison populations in low- and middle-income countries. We searched for studies reporting prevalence rates of nicotine, alcohol, illicit drug, and injection drug use during imprisonment in unselected samples of imprisoned people in low- and middle-income countries. Data meta-analysis was conducted and sources of heterogeneity were examined by meta-regression. Prevalence of nicotine use during imprisonment ranged from 5% to 87%, with a random-effects pooled estimate of 56% (95% confidence interval (CI): 45, 66) with significant geographical heterogeneity. Alcohol use varied from 1% to 76% (pooled prevalence, 16%, 95% CI: 9, 25). Approximately one-quarter of people (25%; 95% CI: 17, 33; range, 0–78) used illicit drugs during imprisonment. The prevalence of injection drug use varied from 0% to 26% (pooled estimate, 1.6%, 95% CI: 0.8, 3.0). Lifetime substance use was investigated in secondary analyses. The high prevalence of smoking in prison suggests that policies regarding smoking need careful review. Furthermore, the findings underscore the importance of timely, scalable, and available treatments for alcohol and illegal drug use by people involved with the criminal justice system.

## INTRODUCTION

Prison populations in low- and middle-income countries (LMICs) have been increasing over the past few decades (1). The increase has been especially pronounced in the Americas and in Oceania. Since the year 2000, prison populations increased by 60% in Oceania, by 80% in Central America, and by 145% in South America (1, 2). Little is known about major causes of morbidity in people involved with the criminal justice system in LMICs, and prison health services rely on evidence from high-income countries. In such settings, a major health problem is substance use disorders (3, 4). These disorders increase the risk of a range of adverse outcomes, including infectious diseases (5), other mental health problems (6), and death (7, 8), and of reoffending on release (9). Although there is high-quality evidence from the general population and prison populations (10) for treatment, there appear to be substantial unmet treatment needs in people involved with the criminal justice system (11). In Latin America, for example, only 1% to 20% of prisons have been reported to have specialized mental health services (11). Bans and treatments in prisons may have continuing effects after release, in contrast to approaches only focusing on forced abstinence in the controlled prison environment (10, 12).

Imprisonment in LMICs is characterized by low budgets that permit providing only basic services, and by overcrowding and human rights violations (11, 13, 14). Human rights concerns have been raised particularly for people with substance use and other psychiatric disorders in prisons (15). In addition to the lack of basic care, there has been little mental health research in prison populations in LMICs (16); such research could assist in providing an evidence base from which to develop services. Based on limited research, it has been suggested that there is a higher prevalence of mental disorders in imprisoned LMIC populations (17); to our knowledge, however, substance use disorders have not been systematically reviewed. This study aims to present a systematic review and meta-analysis of substance use problems in imprisoned people of LMICs while they are in custody and, secondarily, to determine lifetime substance abuse rates.

## METHODS

This systematic review followed the Meta-analysis of Observational Studies in Epidemiology guidelines (18) and data are reported according to the Preferred Reporting Items for Systematic Reviews and Meta-Analyses (19).

### Search strategy

A systematic search of the literature was conducted covering the time from 1987, when the distinction betweenanalytical classification of countries in low-, middle-, and high-income economies was introduced by the World Bank as a development indicator (www.worldbank.org), until March 2017. The search included 1) online databases (i.e., CAB Abstracts; Embase; Global Health; MEDLINE; PsycINFO; Applied Social Sciences Index and Abstracts; Criminal Justice Database; International Bibliography of the Social Sciences; PAIS Index; Social Services Abstracts; LILACS; and Scopus); 2) key journals (e.g., *Addiction*; *Addictive Behaviors*), 3) reference lists of identified papers and relevant systematic reviews; and 4) ProQuest Dissertations & Theses Global, Open Grey, and correspondence with authors. For the online database searches, we used a combined strategy of free-text strings and subject headings related to substance use, prison settings, and prevalence studies (detailed search terms and strings are shown in Web Appendix 1, available at https://academic.oup.com/aje; the results for each online database are shown in Web Table 1, and those for the grey literature are listed in Web Table 2). Non-English articles were translated.

**Figure 1.**
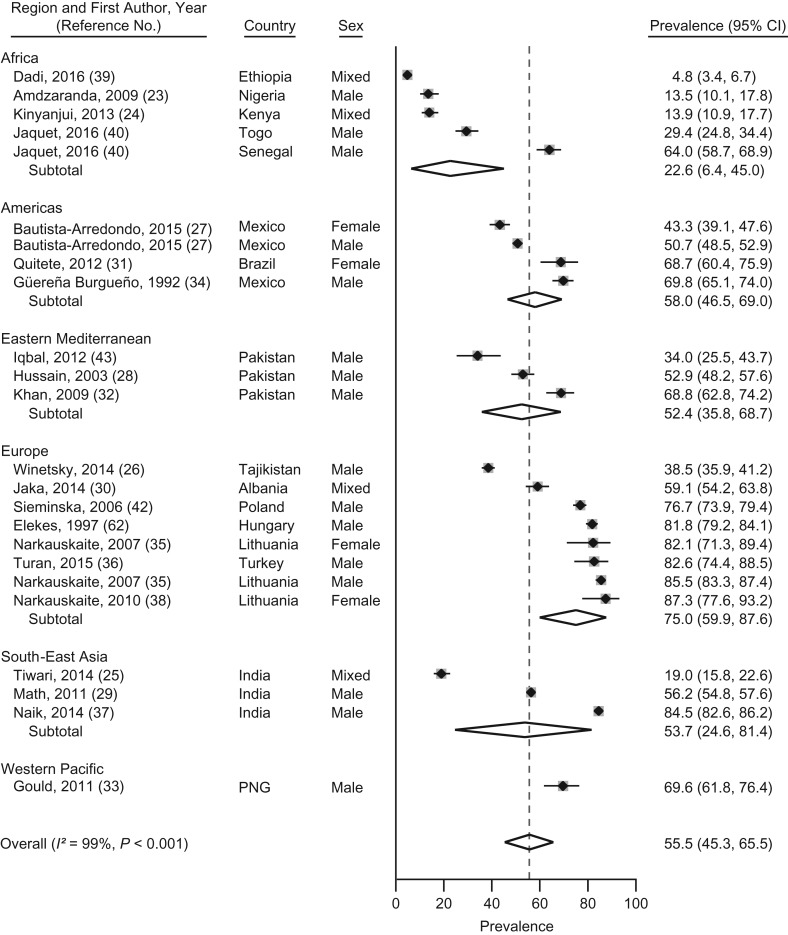
Prevalence and random-effects meta-analyses of nicotine use during imprisonment in low- and middle-income countries by regions as defined by the World Health Organization, 1987–2017. The dashed line indicates the overall pooled random-effects prevalence. CI, confidence interval; PGN, Papua New Guinea.

### Inclusion and exclusion criteria

We identified studies in which prevalence rates were reported of substance use in the general prison population. The following inclusion criteria were applied: 1) Data were collected from unselected general prison populations; 2) the prevalence rates of the use of nicotine, alcohol, illicit drugs, cannabis, cocaine, opiates, and/or injection drugs were established with questionnaires or as part of a research interview; 3) the sampling was representative for the prison population or the population of a facility; and 4) the study was conducted in an LMIC at the time of data collection.

The following exclusion criteria were applied: 1) studies in which a particular age group was selected, such as adolescents or a particular offender type; 2) publications reporting data from the same samples as other publications (the most comprehensive publication was retained); 3) convenience sampling; 4) studies only reporting the prevalence of substance use, applying the disorder criteria (and not the prevalence of substance use without necessarily fulfilling disorder criteria); and 5) studies reporting data collected before 1987 (Web Figure 1).

### Data extraction

Two reviewers (G.B., C.G.) independently extracted the data from the included studies. The following data were extracted: sex, mean age, year and country of data collection, sample size, nonresponse rate, type of substance use, and number of people with any specific type of substance use. The periods covered by the reported prevalence estimates were extracted and coded as during imprisonment (i.e., point prevalence, including ≤1 year) and before imprisonment (including 1 year before imprisonment up to lifetime). When data were missing or clarification was needed, authors of primary studies were contacted. We included people on remand (i.e., in jails, pretrial, and detainees) and sentenced individuals. If the prevalence of heroin use was reported in addition to the prevalence of other opiate use, rates were added to infer the overall prevalence of opiate use. If the prevalence of heroin use was reported as being part of the group of opiate use or vice versa, the higher rate was extracted as the overall prevalence of opiate use. For countries in Europe, Asia, and Africa that did not report opiate use but did report injection drug use, the latter was taken as a proxy for opiate use as well.

### Statistical analysis

If publications reported prevalence estimates separately for men and women or for samples from different countries, they were included in the statistical analyses as different samples. As a consequence, the number of samples is higher than the number of studies. Studies in which less than 10% of participants were of 1 sex were considered representative for the other sex. Separate meta-analyses were conducted for the rates before and during imprisonment. To account for high heterogeneity between the samples, we used random-effects models to balance the weighting of studies for data syntheses (20). To allow comparison between random- and fixed-effects models, fixed-effects meta-analyses also were calculated. Wilson’s method was used to calculate 95% confidence intervals for prevalence estimates (21). Heterogeneity among studies was estimated based on Cochran *Q* test and reported using the *I*^2^ statistic and 95% confidence interval. *I*^2^ > 75% indicated high heterogeneity (22).

Random-effects meta-regressions were conducted to assess the effects of prespecified sample characteristics on the prevalence of substance use. In additional secondary analyses, we included rates of substance use before imprisonment as variables in the regression analyses when assessing the heterogeneity of substance use during imprisonment. Ratios of pooled random-effects prevalence estimates before and during imprisonment were calculated. Ratios (and 95% confidence intervals) of the prevalence in the prison population to the prevalence of the sex-matched general population were calculated for nicotine use because data from the general population were available for most countries. Statistical analyses were conducted with Stata, version 13 (StataCorp LP, College Station, Texas), using the commands *metaprop* for meta-analyses, *metareg* for meta-regressions, and *metan* for the prevalence ratios.

## RESULTS

In 83 studies (n = 94 samples), prevalence rates were reported for substance use by 89,667 individuals who were imprisoned in 32 LMICs within 6 regions as defined by the World Health Organization: Africa, Americas, Eastern Mediterranean, Europe, Southeast Asia, and Western Pacific (Web Figure 2).

### Nicotine use

We identified 24 samples from 17 LMICs reporting prevalence data on nicotine use during imprisonment (23–43). The prevalence of nicotine use during imprisonment ranged from 5% in Ethiopia to 87% in Lithuania. The heterogeneity among the studies was very high (*I*^2^ = 99%; *P* < 0.01). The prevalence was 56% (95% confidence interval (CI): 45, 66), according to pooled random-effects models, among people imprisoned in LMICs (Figure 1). Prevalence of nicotine use was higher in the Americas (58%; β = 0.327; *P* = 0.03) and in Europe (75%; β = 0.483; *P* = 0.001) as compared with Africa, according to meta-regression (Web Table 3).

### Alcohol use

**Figure 2.**
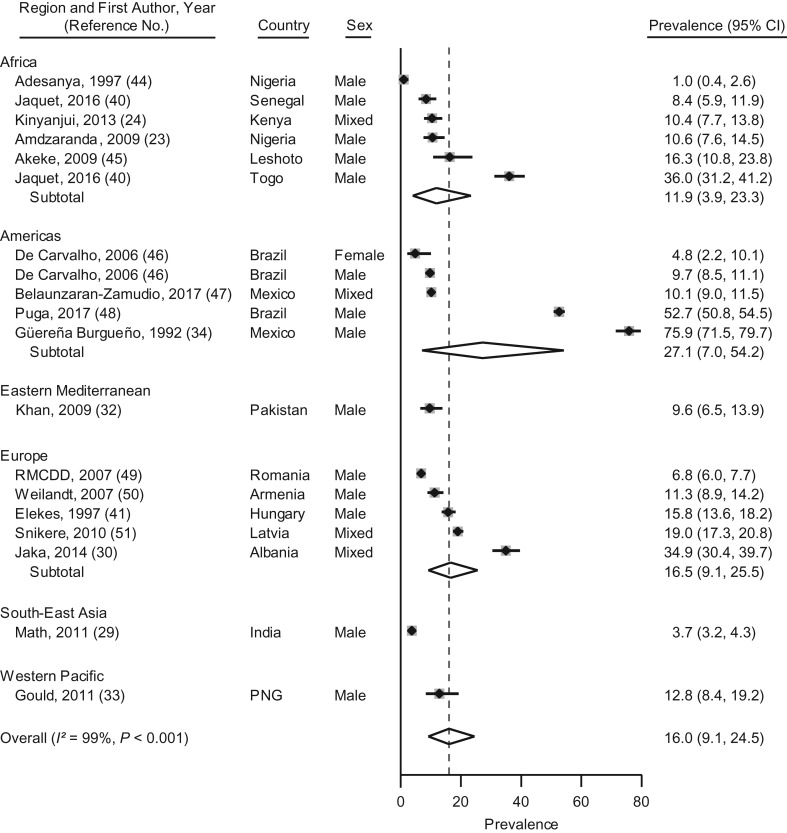
Prevalence and random-effects meta-analyses of alcohol use during imprisonment in low- and middle-income countries by regions as defined by the World Health Organization, 1987–2017. The dashed line indicates the overall pooled random-effects prevalence. CI, confidence interval; PGN, Papua New Guinea.

Alcohol use during imprisonment was reported in 19 samples from 15 LMICs (23, 24, 29, 30, 32–34, 40, 41, 44–51). Prevalence ranged from 1% to 76%; between-study heterogeneity was very high (*I*^2^ = 99%; *P* < 0.01). The random-effects pooled prevalence was 16% (95% CI: 9, 25) (Figure 2). Meta-regression analyses did not show any associations between predetermined study characteristics and alcohol use during imprisonment (Web Table 4).

### Illicit drug use during imprisonment

**Figure 3.**
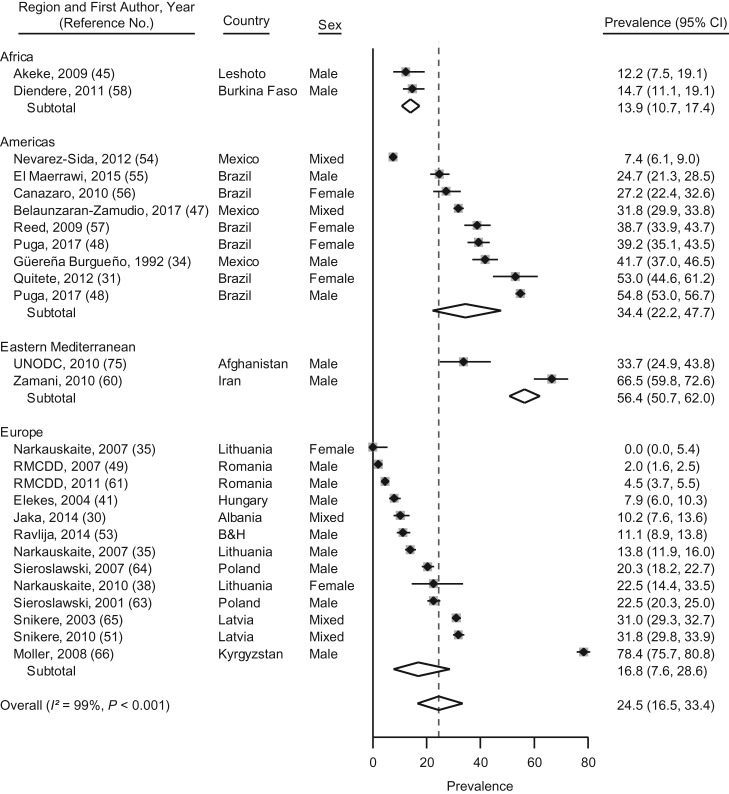
Prevalence and random-effects meta-analyses of illicit drug use during imprisonment in low- and middle-income countries by regions as defined by the World Health Organization, 1987–2017. The dashed line indicates the overall pooled random-effects prevalence. B&H, Bosnia and Herzegovina; CI, confidence interval.

There were 26 samples from 14 LMICs reporting prevalence estimates for any illicit drug use during imprisonment (30, 31, 34, 35, 38, 45, 47–49, 51–65). These estimates ranged from 0% for imprisoned women in Lithuania to 78% for male prisoners in Kyrgyzstan (*I*^2^ = 99%; *P* < 0.01) (Figure 3). Random-effects pooled prevalence was 25% (95% CI: 17, 33) (Figure 3). No significant associations between study characteristics were found on meta-regression analysis (Web Table 5).

#### Cannabis use

Prevalence estimates of cannabis use during imprisonment were reported for 30 samples in 16 LMICs (23, 24, 27, 31, 32, 34, 40, 41, 43, 44, 46, 47, 50, 51, 53, 56–58, 60–66); these varied from 1% to 55% (Web Figure 3), with high heterogeneity (*I*^2^ = 99%; *P* < 0.01). Pooled prevalence on random-effects meta-analysis was 17% (95% CI: 12, 23) for cannabis use during imprisonment in LMICs. In the Americas, cannabis use was more frequent (25%; β = 0.145; *P* = 0.041) than in Africa (Web Table 6).

#### Other drugs

Prevalence estimates of cocaine use during imprisonment were reported for 20 samples in 8 LMICs (23, 27, 31, 32, 34, 41, 44, 46–48, 51, 53, 56, 60–64). Prevalence estimates for cocaine use during imprisonment ranged from 0% to 29% (Web Figure 4). The heterogeneity between the studies was high (*I*^2^ = 99.2%; *P* < 0.001). According to pooled rates, the prevalence of cocaine use during imprisonment in LMICs was 5% (95% CI: 2, 8). On meta-regression, no study characteristics were associated with the prevalence of cocaine use during imprisonment (Web Table 7).

Prevalence estimates of opiate use during imprisonment were reported for 26 samples in 14 LMICs (23, 32, 34, 35, 41, 43, 44, 47, 50–52, 58–60, 62–65, 67–71); these rates varied from 0% to 80% (Web Figure 5), with a pooled prevalence of 6% (95% CI: 3, 11). No associations were found on meta-regression (Web Table 8).

#### Injection drug use during imprisonment

**Figure 4.**
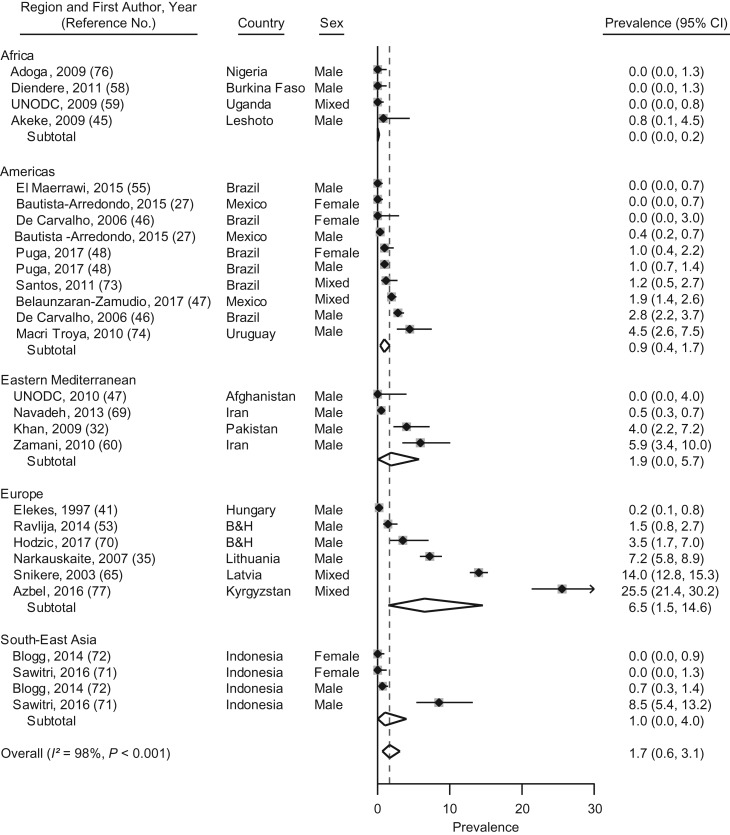
Prevalence and random-effects meta-analyses of injection drug use during imprisonment in low- and middle-income countries by regions as defined by the World Health Organization, 1987–2017. The dashed line indicates the overall pooled random-effects prevalence. B&H, Bosnia and Herzegovina; CI, confidence interval.

Prevalence estimates for injection drug use during imprisonment were reported for 28 samples from 16 LMICs (27, 32, 35, 41, 45–48, 52, 54, 57–59, 64, 68–76); these rates varied from 0% to 26% (Figure 4). The pooled random-effects prevalence was 1.7% (95% CI: 0.6, 3.1). The rate of injection drug use in European LMICs was higher than in Africa, according to meta-regression analyses (6.5%; β = 0.087; *P* = 0.027) (Web Table 9).

### Other analyses

Prevalence rates of substance use before imprisonment are reported in the Web Appendices 2–8 and in the Web Figures 6–12 (24–27, 29, 32–35, 38, 40–42, 44–52, 54, 55, 57–106). Pooled random-effects estimates were 70% for nicotine use (Web Appendix 2 and Web Figure 6), 71% for alcohol use (Web Appendix 3 and Web Figure 7), 48% for illicit drug use (Web Appendix 4 and Web Figure 8), 37% for cannabis use (Web Appendix 5 and Web Figure 9), 23% for cocaine use (Web Appendix 6 and Web Figure 10), 10% for opiate use (Web Appendix 7 and Web Figure 11), and 10% for injection drug use (Web Appendix 8 and Web Figure 12). There was clear geographical heterogeneity for nicotine use, alcohol use, cocaine use, opiate use, and injection drug use before imprisonment (Web Tables 3–9). Web Table 10 lists all included studies reporting the prevalence of substance use in prison populations during or before imprisonment.

We assessed whether substance use before imprisonment explained part of the heterogeneity of substance use during imprisonment. For nicotine, alcohol, any illicit drug use, and injection drug use, the prevalence before imprisonment was not associated with heterogeneity. However, for cannabis (*P* < 0.001), cocaine (*P* < 0.001), and opiate use (*P* < 0.001), the prevalence before imprisonment was significantly associated with the prevalence during imprisonment, according to univariate analyses (Web Tables 3–9). According to multivariate meta-regression, sex (β = 0.198; *P* = 0.050) and imprisonment in Europe (β = 0.236; *P* = 0.009) retained statistical significance for the prevalence of nicotine use before imprisonment (Web Table 3). Only cannabis use before imprisonment (β = 0.00018; *P* < 0.001) remained significant for cannabis use during imprisonment (Web Table 6). Regional heterogeneity for cocaine use before imprisonment was supported by multivariate regression analysis, with higher prevalence in the Americas (β = 0.389; *P* < 0.001) and in Europe (β = 0.104; *P* = 0.031) (Web Table 7). For the prevalence of opiate use before imprisonment, the prevalence in the Eastern Mediterranean region (β = 0.135; *P* < 0.046) and the nonresponse rate (β = 0.0022; *P* = 0.018) were significant in multivariate analyses (Web Table 8). Ratios of pooled random-effects prevalence rates before compared with during imprisonment were 1.3 (95% CI: 1.2, 1.3) for nicotine use; 4.4 (95% CI: 4.3, 4.6) for alcohol use; 2.0 (95% CI: 1.9, 2.0) for any illicit drug use; 2.2 (95% CI: 2.1, 2.3) for cannabis use; 4.9 (95% CI: 4.6, 5.2) for cocaine use; 1.7 (95% CI: 1.6, 1.8) for use of opiates; and 60 (95% CI: 55, 66) for injection drug use. Ratios were consistently higher than 1, indicating higher rates for substance use before than during imprisonment. Fixed-effects models for all analyses are presented in Web Table 11.

**Figure 5.**
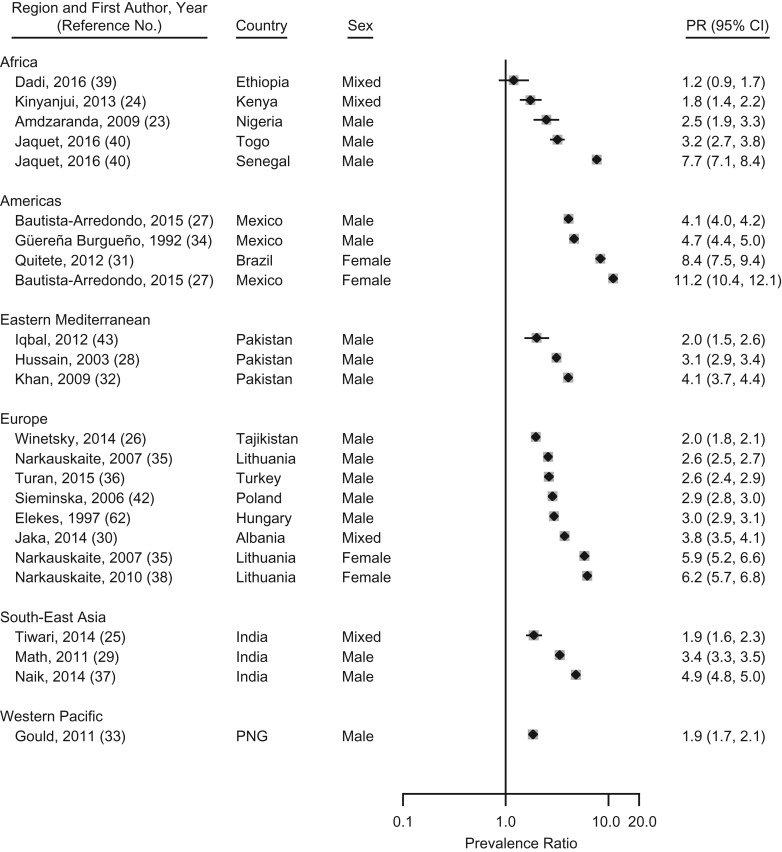
Prevalence ratios of nicotine use during imprisonment to nicotine use in the general population by regions as defined by the World Health Organization, 1987–2017. The dashed line indicates the overall pooled random-effects prevalence. CI, confidence interval; PGN, Papua New Guinea; PR, prevalence ratio.

We estimated prevalence ratios for nicotine use, which were prevalence rates among prison populations compared with sex-matched estimates in the general population from the countries where those prisons were located. For nicotine, prevalence ratios were all higher than 1 and ranged from 1.2 (95% CI: 0.9, 1.7) in Ethiopia to 11.2 (95% CI: 10.4, 12.1) in Mexico (Figure 5).

## DISCUSSION

### Main results

We have provided estimates of nicotine, alcohol, and illicit drug use during imprisonment from a systematic review and meta-analysis of 94 samples in 83 studies and nearly 90,000 people imprisoned in LMICs. There were 3 main findings. First, the pooled random-effects prevalence of nicotine use during imprisonment was 56%; the prevalence ratios of all the included primary studies were higher than those of the general populations. Second, a pooled prevalence of 16% was calculated for alcohol use during imprisonment, with wide variations in geographical prevalence. Third, it was estimated that approximately one-quarter of the imprisoned people (pooled prevalence, 25%, 95% CI: 17, 33) used illicit drugs during imprisonment, and injection drug use during imprisonment was estimated at 1.7% (95% CI: 0.6, 3.1).

### Strength and limitations

To our knowledge, this is the first systematic review of substance use problems in prison populations of LMICs worldwide. We provide a sufficient body of evidence from data synthesis and have conducted meta-regression analyses examining sources of heterogeneity. The pooled prevalence estimates have to be interpreted with caution because of the high between-study heterogeneity of the data, which would be expected, considering the differences in criminal justice systems and prisons around the world. As a consequence, in addition to a random-effects prevalence that assumes heterogeneity, we have provided prevalence ranges and fixed-effects models. The latter weigh studies more by sample size and may be informative when small study effects are considered strong. An additional limitation of this study is the variability of the policy contexts with respect to (partial) smoking bans and the implementation of drug and alcohol bans.

### Implications

In contrast to the other substances reviewed here, nicotine use is legal in prisons in LMICs, to our knowledge. A principal implication of this review is the need to review smoking policies inside prisons in LMICs. Policy initiatives in this regard are relatively new in high-income countries, where tobacco control generally has been more effective (107). Several high-income countries have introduced smoking bans in prisons and jails (108–111), which should reduce morbidity and death among people involved in the criminal justice system (108). Smoking bans during imprisonment combined with psychological interventions before release can be successful in prolonging abstinence from smoking after release (10). In the current meta-analysis, we report more than half of the people imprisoned in LMICs smoked during imprisonment, and thus, the potential for addressing this is considerable. We also have shown that these rates of nicotine use are substantially higher than in the general population of the specific countries of the primary studies included in the review. Therefore, policies to ban smoking in prisons and treatments to reduce nicotine addiction should be considered in LMICs, especially in the Americas and in Europe, where the rates are particularly high.

A second implication is the importance of ensuring that alcohol treatments are available in prisons in LMICs. There has been considerable interest in addressing drug use, particularly as it is associated with infectious diseases, but in this review, we found that approximately 1 in 6 imprisoned people consumes alcohol inside prison. Many of these individuals may not have alcohol use disorders, but many do and will continue to on release. Because alcohol is usually banned in prisons and difficult to smuggle, it is often produced inside prisons and, consequently, tends to be of low quality and high toxicity (33, 112). Prison services need to have available appropriate alcohol detoxification treatments on entry and to consider other interventions, including group therapies and other psychosocial treatments that are scalable (113). In addition, the heterogeneity of prevalence estimates for alcohol use during imprisonment indicates the need for local surveys to best inform service development. Such local surveys may not be feasible owing to financial pressures in some countries; therefore, the estimates presented here could be of assistance.

A third implication is that the findings underscore the importance of addressing illicit drug use during imprisonment. High-income countries have high rates and persistence of illicit drug use during imprisonment, especially heroin use (114). We found that approximately one-quarter of the prison population uses illicit drugs during imprisonment. There is good evidence for the effectiveness of opioid-substitution treatment in prison populations (115). However, apart from initiatives in a few countries (116), treatment interventions are mostly unavailable in LMICs. In addition, we report important regional differences in the prevalence of cannabis and possibly of opiate and cocaine use. This would suggest that rather than 1 treatment model for all countries, interventions may need to be tailored at regional or national levels for specific types of drug use problems.

Injection drug use is a risk factor for spreading HIV in prisons in LMICs (117, 118). There remains a paucity of data available from LMICs (117), with data from 32 countries and considerable heterogeneity in the findings. More research on the changing dynamics of injection drug use in LMICs is required.

Individual prevalence rates of specific types of illicit drugs used before imprisonment, including cannabis, opiates, and cocaine, explained part of the heterogeneity in illicit drug use prevalence during imprisonment. We also found that prevalence rates of substance use before imprisonment were consistently higher than those during imprisonment for all substances. This difference was more pronounced for alcohol than for illicit drugs, suggesting that prison systems more effectively limit alcohol than illicit drugs. Overall, this suggests that people import their substance use problems into prison and further underscores the need for intervention programs to be integrated between the community and prison.

### Conclusion

We report estimates of smoking, alcohol, and illicit drug use during imprisonment in LMICs. Approximately 1 in 2 prisoners smokes, 1 in 6 drinks alcohol, and 1 in 4 uses illicit drugs. From a public health perspective, these high rates represent an opportunity for intervention, particularly because interventions that are effective in other settings can be transferred to prisons. Smoking bans in prisons, and scalable and available detoxification and addiction services have the potential to address the large burdens of smoking and substance use in LMICs.

## Supplementary Material

Supplementary Data

## Abbreviations

CI: confidence interval
LMIC: low- and middle-income country
