# Supplementary material for: Substance Use During Imprisonment in Low- and Middle-Income Countries
Source: Epidemiol Rev. 2018 Mar 23;40(1):70–81. doi: 10.1093/epirev/mxx016 (PMC5982797; doi:10.1093/epirev/mxx016)
Supplement: Supplementary Data [file mxx016mundtwebmaterialrevised.pdf]

Supplementary data

Substance use during imprisonment in low- and middle-income countries

Web Appendix 1. General search string

Prison condition:

Prison* OR imprison* OR sentence* OR remand* OR jail* OR correctional OR "criminal justice"

Epidemiological studies:

Epidemiology (Subject Heading) OR prevalence OR incidence

Mental health outcome:

Mental Disorders (Subject Heading) OR Substance-Related Disorders (Subject Heading) OR psychiatr* OR psycholog* OR smok* OR alcohol* OR substance OR drug OR addict* OR inject*

Limit: 1987 to March 2017

Web Table 1. Database searches

SOURCES (11)		
**Prison**	**Epidemiology**	**Mental health**
**Applied Social Sciences Index and Abstracts (ASSIA) (24/3/2017) (516)***		
psychiatr* OR psycholog* OR smok* OR alcohol* OR substance OR drug OR addict* OR inject* (227,050)	prevalence OR incidence OR su.Exact("epidemiology") (30,037)	(su.Exact("substance-related disorders") OR su.Exact("substance abuse" OR "substance abuse disorders")) OR (psychiatr* OR psycholog* OR smok* OR alcohol* OR substance OR drug OR addict* OR inject*) (17,392)
**CAB Abstracts *1973 to 2017 Week 11* (24/3/2017) (225)***		
(Prison* or imprison* or sentence* or remand* or jail* or correctional or criminal justice).mp (2,003)	epidemiology/ or (prevalence or incidence).mp. (422,950)	mental disorders/ or substance abuse/ or (psychiatr* or psycholog* or smok* or alcohol* or substance or drug or addict* or inject*).mp. (792,500)
**Criminal Justice Database (24/3/2017) (607)***		
all(Prison* OR imprison* OR sentence* OR remand* OR jail* OR correctional OR "criminal justice") (135,926)	all(prevalence OR incidence) OR su.Exact("epidemiology") (13,226)	su.Exact("mental disorders") OR su.Exact("substance-related disorders") OR all(psychiatr* OR psycholog*

		OR smok* OR alcohol* OR substance OR drug OR addict*OR inject*) (149,574)
**Embase** ***1980 to 2017 Week 12*** **(24/3/2017) (3518)***		
(Prison* or imprison* or sentence* or remand* or jail* or correctional or criminal justice).mp. (55,127)	epidemiology/ or (prevalence or incidence).mp. (1,863,770)	mental disease/ or substance abuse/ or "substance use"/ or (psychiatr* or psycholog* or smok* or alcohol* or substance or drug or addict* or inject*).mp. (9,217,897)
**Global Health** ***1973 to 2017 Week 11*** **(24/3/2017) (857)***		
(Prison* or imprison* or sentence* or remand* or jail* or correctional or criminal justice).mp (3,264)	epidemiology/ or (prevalence or incidence).mp. (298,177)	mental disorders/ or substance abuse/ or (psychiatr* or psycholog* or smok* or alcohol* or substance or drug or addict* or inject*).mp. (503,739)
**International Bibliography of the Social Sciences (IBSS) (24/3/2017) (1098)***		
Prison* OR imprison* OR sentence* OR remand* OR jail* OR correctional OR “criminal justice” (37,180)	su.Exact("epidemiology") OR (prevalence OR incidence) (21,074)	su.Exact("mental disorders") OR su.Exact("substance abuse" OR "substance use") OR (psychiatr* OR psycholog* OR smok* OR alcohol* OR substance OR drug OR addict* OR inject*) (172,839)
**LILACS (4/4/2017) (41)***		
prison$ OR imprison$ OR sentence$ OR remand$ OR jail$ OR correctional OR criminal justice [Words] (1362)	epidemiolog$ OR prevalence OR incidence [Words] (108,772)	mental OR substance OR psychiatr$ OR psycholog$ OR smok$ OR alcohol$ OR substance OR drug OR addict$ OR inject$ [Words] (136,139)
**MEDLINE*****(R) Epub Ahead of Print, In-Process & Other Non-Indexed Citations, Ovid MEDLINE(R) Daily and Ovid MEDLINE(R) 1946 to Present*** **(24/3/2017) (2406)***		
(Prison* or imprison* or sentence* or remand* or jail* or correctional or criminal justice).mp. (46,512)	Epidemiology/ or (prevalence or incidence).mp. (1,234,033)	Mental Disorders/ or Substance-Related Disorders/ or (psychiatr* or psycholog* or smok* or alcohol* or substance or drug or addict* or inject*).mp. (4,241,016)
**PAIS Index (3/4/2017) (93)***		
prison* OR imprison* OR sentence* OR remand* OR jail* OR correctional OR “criminal justice” (17,806)	su.Exact("epidemiology") OR (prevalence OR incidence) (6,750)	su.Exact("mental disorders") OR su.Exact("substance abuse") OR (psycholog* OR smok* OR alcohol* OR substance OR drug OR addict* OR inject*) (36,460)
**PsycINFO** ***1806 to March Week 3 2017*** **(24/3/2017) (1620)***		
(Prison* or imprison* or sentence* or remand* or jail*	epidemiology/ or (prevalence or incidence).mp. [mp=title, abstract, heading word, table of	mental disorders/ or "substance use disorder"/ or (psychiatr* or psycholog* or smok* or

or correctional or criminal justice).mp. (73,554)	contents, key concepts, original title, tests & measures] (149,748)	alcohol* or substance or drug or addict*OR inject*).mp. (1,176,019)
**Scopus (24/3/2017) (4,580)***		
TITLE-ABS-KEY (prison* OR imprison* OR sentence* OR remand* OR jail* OR correctional OR "criminal justice") (141,178)	(TITLE-ABS-KEY (epidemiolog* OR prevalence OR incidence) (2,158,208)	TITLE-ABS-KEY (mental OR substance OR psychiatr* OR psycholog* OR smok* OR alcohol* OR substance OR drug OR addict* OR inject*) (10,794,492)
**Social Services Abstracts (24/3/2017) (197)***		
Prison* OR imprison* OR sentence* OR remand* OR jail* OR correctional OR "criminal justice" (6,532)	su.Exact("epidemiology") OR (prevalence OR incidence) (8,922)	su.Exact("substance-related disorders" OR "substance abuse" OR "substance abuse disorders") OR su.Exact("mental disorders") OR (psychiatr* OR psycholog* OR smok* OR alcohol* OR substance OR drug OR addict* OR inject*) (56,065)

**Number after discarding duplicates*

Web Table 2. Grey Literature

SOURCES (2)		
**Prison**	**Epidemiology**	**Mental Health**
**Open Grey (03/4/2017) (25)†**		
prison* OR imprison* OR sentence* OR remand* OR jail* OR correctional OR "criminal justice" (3587)	epidemiolog* OR prevalence OR incidence (14,287)	mental OR substance OR psychiatr* OR psycholog* OR smok* OR alcohol* OR substance OR drug OR addict* OR inject* (80,467)
**ProQuest Dissertations & Theses Global (3/4/2017) (375)***		
all(prison* OR imprison* OR sentence* OR remand* OR jail* OR correctional OR "criminal justice") (33,981)	all(prevalence OR incidence) OR su.Exact("epidemiology") (68,101)	su.Exact("drug abuse" OR "drug use" OR "mental disorders") OR all(psycholog* OR smok* OR alcohol* OR substance OR drug OR addict* OR inject*) (593,744)

**Number after discarding duplicates*

†Hits were not exported in reference manager

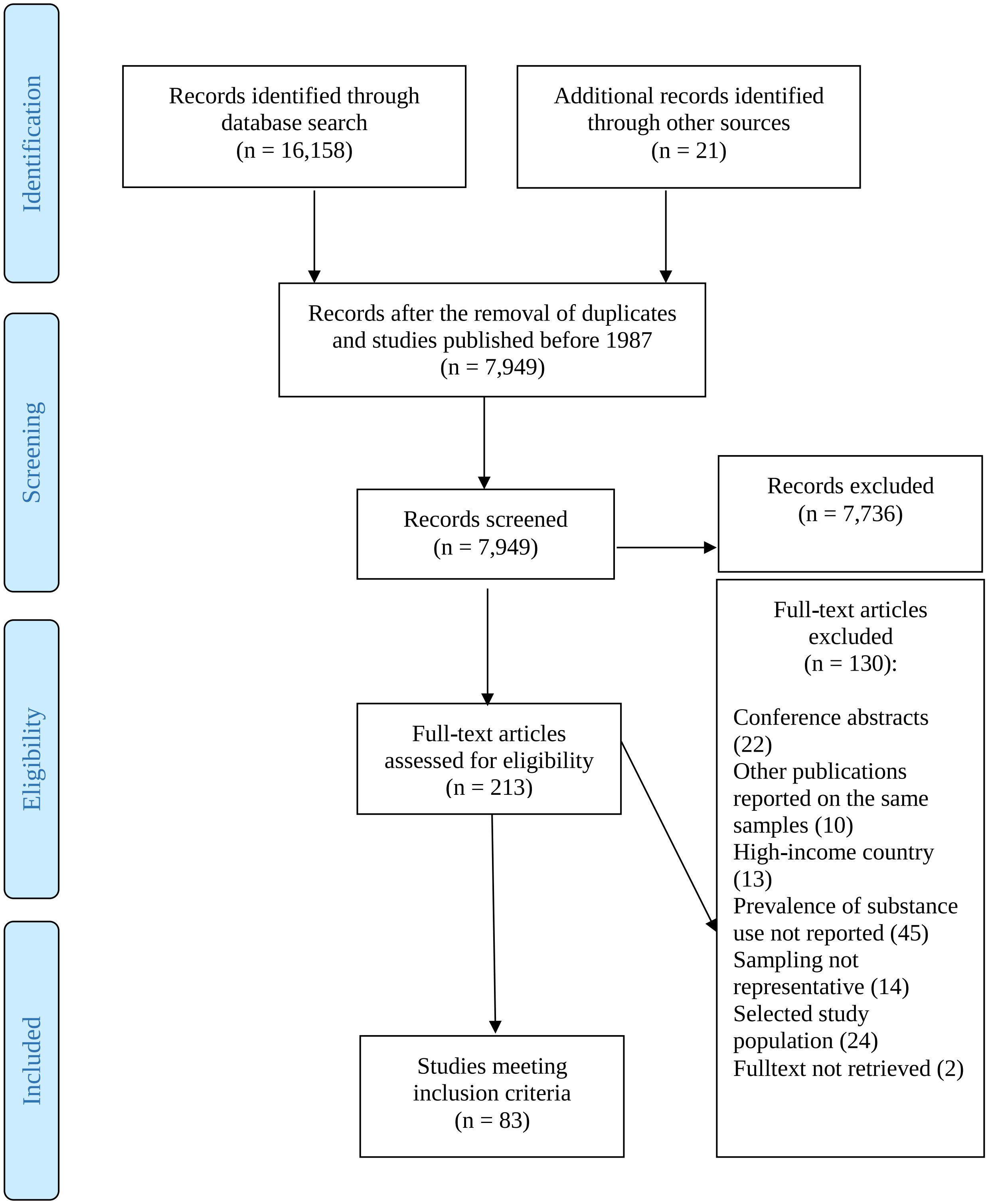

Web Figure 1. PRISMA Flow-Chart of studies identified, screened and included in the systematic review on the prevalence of substance use in prison populations in low- and middle-income countries between 1987 and 2017.

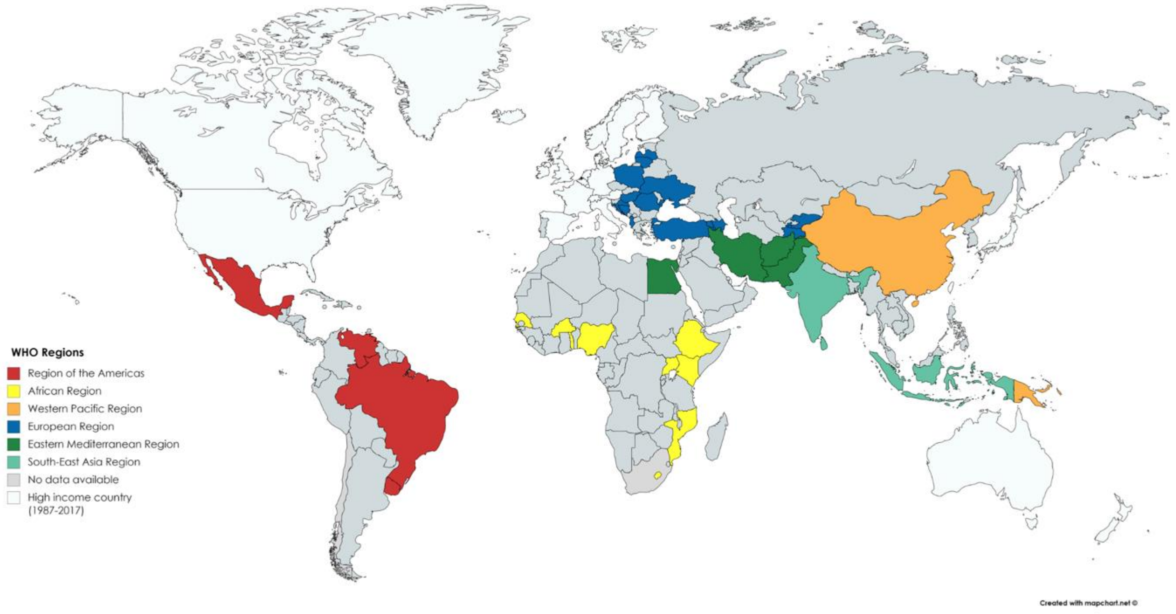

Web Figure 2. Map of low- and middle-income countries with data reporting substance use of imprisoned people by WHO-regions. Light grey are countries that have been in the high-income category since the implementation of the categories in 1987. Dark grey represents low- and middle-income countries without identified data.

Web Table 3. Meta-regression analyses of study characteristics and the prevalence of nicotine use among people who are imprisoned in low- and middle-income countries.

**Substance**	**Nicotine use**							
**Prevalence period**	***Before imprisonment***				***During imprisonment***			
	Coefficient (ß)	95% CI lower	95% CI upper	P	Coefficient (ß)	95% CI lower	95% CI upper	P
Sex	0.209	0.015	0.403	**0.036**	-0.086	-0.353	0.181	0.510
Age	-0.009	-0.044	0.027	0.612	0.001	-0.032	0.035	0.927
Sample size	2.94e-06	-0.0001	0.0001	0.930	0.00001	-0.0001	0.0001	0.844
Non-response rate	0.001	-0.003	0.005	0.582	0.003	-0.001	0.007	0.167
Before imprisonment	n.a.				0.00006	-0.00013	0.00025	0.490
WHO-regions (Africa Reference category)								
Americas	0.086	-0.129	0.300	0.408	0.327	0.035	0.619	**0.030**
Eastern Mediterranean	0.210	-0.012	0.432	0.062	0.277	-0.046	0.600	0.068
Europe	0.278	0.082	0.474	**0.009**	0.484	0.234	0.734	**0.001**
South East Asia	0.088	-0.160	0.336	0.463	0.286	-0.027	0.600	0.071
Western Pacific	0.186	-0.049	0.420	0.112	0.250	-0.048	0.941	0.074
Multivariate meta-regression analyses retaining significant variables								
Sex	0.198	0.000	0.386	**0.050**				
Europe	0.236	0.069	0.402	**0.009**				

Web Table 4. Meta-regression analyses of study characteristics and the prevalence of alcohol use among people who are imprisoned in low- and middle-income countries.

**Substance**	**Alcohol use**							
**Prevalence period**	***Before imprisonment***				***During imprisonment***			
	Coefficient (ß)	95% CI lower	95% CI upper	P	Coefficient (ß)	95% CI lower	95% CI upper	P
Sex	0.166	-0.031	0.364	0.094	0.145	-0.320	0.612	
Age	-0.003	-0.043	0.037	0.879	-0.016	-0.051	0.019	0.345
Sample size	-0.000013	-0.0001	0.00005	0.680	-0.00001	-0.00008	0.00006	0.707
Non-response rate	0.003	-0.002	0.008	0.174	-0.002	-0.010	0.005	0.546
Before imprisonment	n.a.				-0.00001	-0.00006	0.00005	0.750
WHO-regions (Africa Reference category)								
Americas	-0.012	-0.159	0.134	0.864	0.174	-0.089	0.437	0.178
Eastern Mediterranean	-0.278	-0.476	-0.079	**0.009**	-0.041	-0.517	0.435	0.855
Europe	0.151	-0.002	0.303	**0.052**	0.037	-0.225	0.299	0.765
South East Asia	-0.179	-0.420	0.063	0.137	-0.100	-0.557	0.357	0.644
Western Pacific	0.081	-0.107	0.269	0.378	-0.009	-0.498	0.481	0.970

Web Table 5. Meta-regression analyses of study characteristics and the prevalence of illicit drug use among people who are imprisoned in low- and middle-income countries.

**Substance**	**Illicit drug use**							
**Prevalence period**	***Before imprisonment***				***During imprisonment***			
	Coefficient (ß)	95% CI lower	95% CI upper	P	Coefficient (ß)	95% CI lower	95% CI upper	P
Sex	0.058	-0.095	0.211	0.449	-0.041	-0.254	0.172	0.693
Age	-0.016	-0.048	0.016	0.318	0.003	-0.059	0.064	0.927
Sample size	-0.000061	-0.00012	-1.22e-06	0.046	-0.000012	-0.000099	0.0000751	0.772
Non-response rate	0.002	-0.0009	0.005	0.193	-0.003	-0.006	0.0006	0.108
Before imprisonment	n.a.				0.00010	-0.00013	0.00034	0.373
WHO-regions (Africa Reference category)								
Americas	0.185	-0.050	0.420	0.119	0.216	-0.094	0.527	0.163
Eastern Mediterranean	0.164	-0.097	0.424	0.213	0.378	-0.028	0.784	0.066
Europe	-0.095	-0.326	0.137	0.416	0.066	-0.236	0.367	0.653
South East Asia	-0.161	-0.453	0.131	0.272	Dropped	-	-	-
Western Pacific	-0.272	-0.567	0.023	0.070	Dropped	-	-	-

Region and First Author, Year (Reference No.)	Country	Sex	PR (95% CI)
Africa			
Kinyanjui, 2013 (24)	Kenya	Mixed	5.1 (3.3, 7.7)
Adesanya, 1997 (44)	Nigeria	Male	6.6 (4.5, 9.5)
Amdzaranda, 2009 (23)	Nigeria	Male	6.9 (4.6, 10.4)
Jaquet, 2016 (40)	Togo	Male	7.5 (5.2, 10.8)
Jaquet, 2016 (40)	Senegal	Male	7.5 (5.1, 10.8)
Diendere, 2011 (57)	Burkina Faso	Male	12.7 (9.4, 16.9)
Vaz, 1995 (66)	Mozambique	Female	16.7 (9.0, 28.7)
Vaz, 1995 (66)	Mozambique	Male	29.5 (27.1, 32.1)
Subtotal			10.6 (4.6, 18.8)
Americas			
Nevarez-Sida, 2012 (53)	Mexico	Mixed	5.4 (4.3, 6.8)
Bautista-Arredondo, 2015 (27)	Mexico	Female	10.0 (7.7, 12.9)
De Carvalho, 2006 (46)	Brazil	Female	15.2 (10.0, 22.5)
Bautista-Arredondo, 2015 (27)	Mexico	Male	23.8 (21.9, 25.7)
Belaunzaran-Zamudio, 2017 (47)	Mexico	Mixed	25.8 (24.0, 27.7)
De Carvalho, 2006 (46)	Brazil	Male	27.6 (25.6, 29.6)
Reed, 2009 (56)	Brazil	Female	32.1 (27.6, 37.0)
Güereña Burgueño, 1992 (34)	Mexico	Male	33.9 (29.5, 38.6)
Quitete, 2012 (31)	Brazil	Female	41.8 (33.8, 50.3)
Puga, 2017 (48)	Brazil	Male	45.9 (44.1, 47.8)
Subtotal			24.9 (16.5, 34.3)
Eastern Mediterranean			
Iqbal, 2012 (43)	Pakistan	Male	3.0 (1.0, 8.5)
Khan, 2009 (32)	Pakistan	Male	28.0 (22.8, 33.9)
UNODC, 2010 (58)	Afghanistan	Male	28.3 (20.1, 38.2)
Subtotal			17.7 (3.8, 38.3)
Europe			
RMCDD, 2011 (60)	Romania	Male	0.8 (0.5, 1.3)
Elekes, 1997 (41)	Hungary	Male	2.0 (1.3, 3.1)
Elekes, 2004 (61)	Hungary	Male	6.2 (4.6, 8.4)
Weilandt, 2007 (50)	Armenia	Male	7.4 (5.5, 9.9)
Sieroslawski, 2007 (63)	Poland	Male	18.3 (16.3, 20.6)
Sieroslawski, 2001 (62)	Poland	Male	21.3 (19.0, 23.7)
Snikere, 2010 (51)	Latvia	Mixed	24.2 (22.4, 26.2)
Snikere, 2003 (64)	Latvia	Mixed	28.0 (26.4, 29.7)
Moller, 2008 (65)	Kyrgyzstan	Male	55.1 (52.0, 58.1)
Subtotal			15.1 (6.0, 27.4)
Overall (I^2 = 99%, P < 0.001)			17.1 (12.0, 22.9)

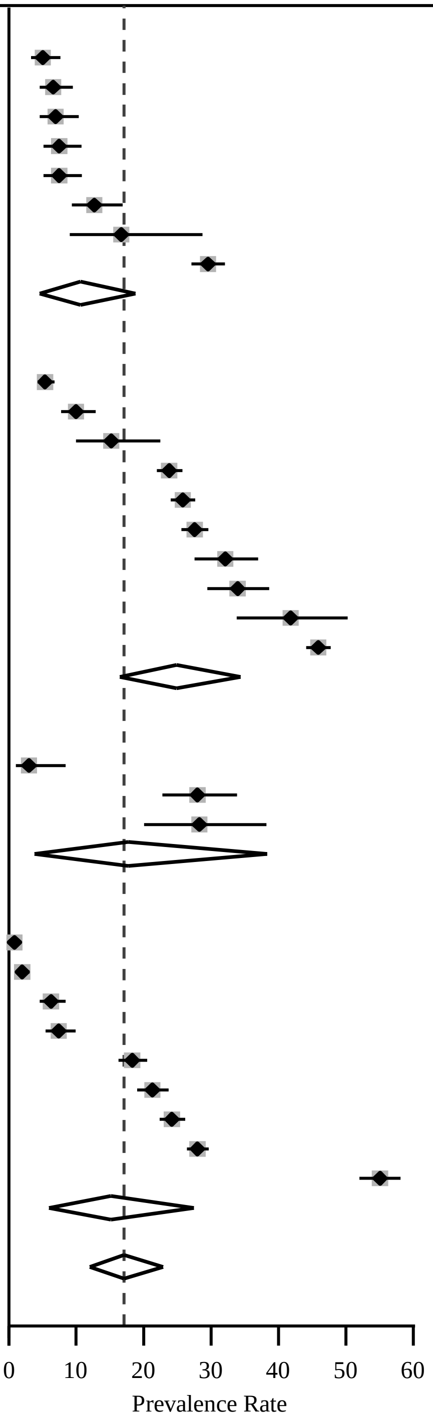

Web Figure 3. Prevalence rates and random effects meta-analyses of cannabis use during imprisonment in low- and middle-income countries by regions as defined by the World Health Organization, 1987-2017. CI, confidence interval; PR, prevalence rate.

Web Table 6. Meta-regression analyses of study characteristics and the prevalence of cannabis use among people who are imprisoned in low- and middle-income countries.

**Substance**	**Cannabis use**							
**Prevalence period**	***Before imprisonment***				***During imprisonment***			
	Coefficient (ß)	95% CI lower	95% CI upper	P	Coefficient (ß)	95% CI lower	95% CI upper	P
Sex	-0.157	-0.338	0.024	0.086	-0.043	-0.206	0.120	0.589
Age	-0.014	-0.069	0.041	0.605	-0.003	-0.024	0.018	0.779
Sample size	-0.0000431	-0.0001	0.00002	0.161	0.0000525	-9.43e-06	0.0001144	0.094
Non-response rate	0.004	-0.0001	0.009	0.056	0.002	-0.002	0.006	0.396
Before imprisonment	n.a.				0.00019	0.00013	0.00024	<**0.001**
WHO-regions (Africa Reference category)								
Americas	0.193	-0.005	0.391	0.056	0.145	0.007	0.284	**0.041**
Eastern Mediterranean	Dropped	-	-	-	0.087	-0.127	0.304	0.405
Europe	0.009	-0.189	0.206	0.928	0.067	-0.073	0.207	0.331
South East Asia	-0.185	-0.541	0.171	0.291	Dropped	-	-	-
Western Pacific	Dropped	-	-	-	Dropped	-	-	-
Multivariate meta-regression analyses retaining significant variables								
Before imprisonment					0.00018	0.00012	0.00023	<**0.001**
Americas					0.036	-0.054	0.126	0.404

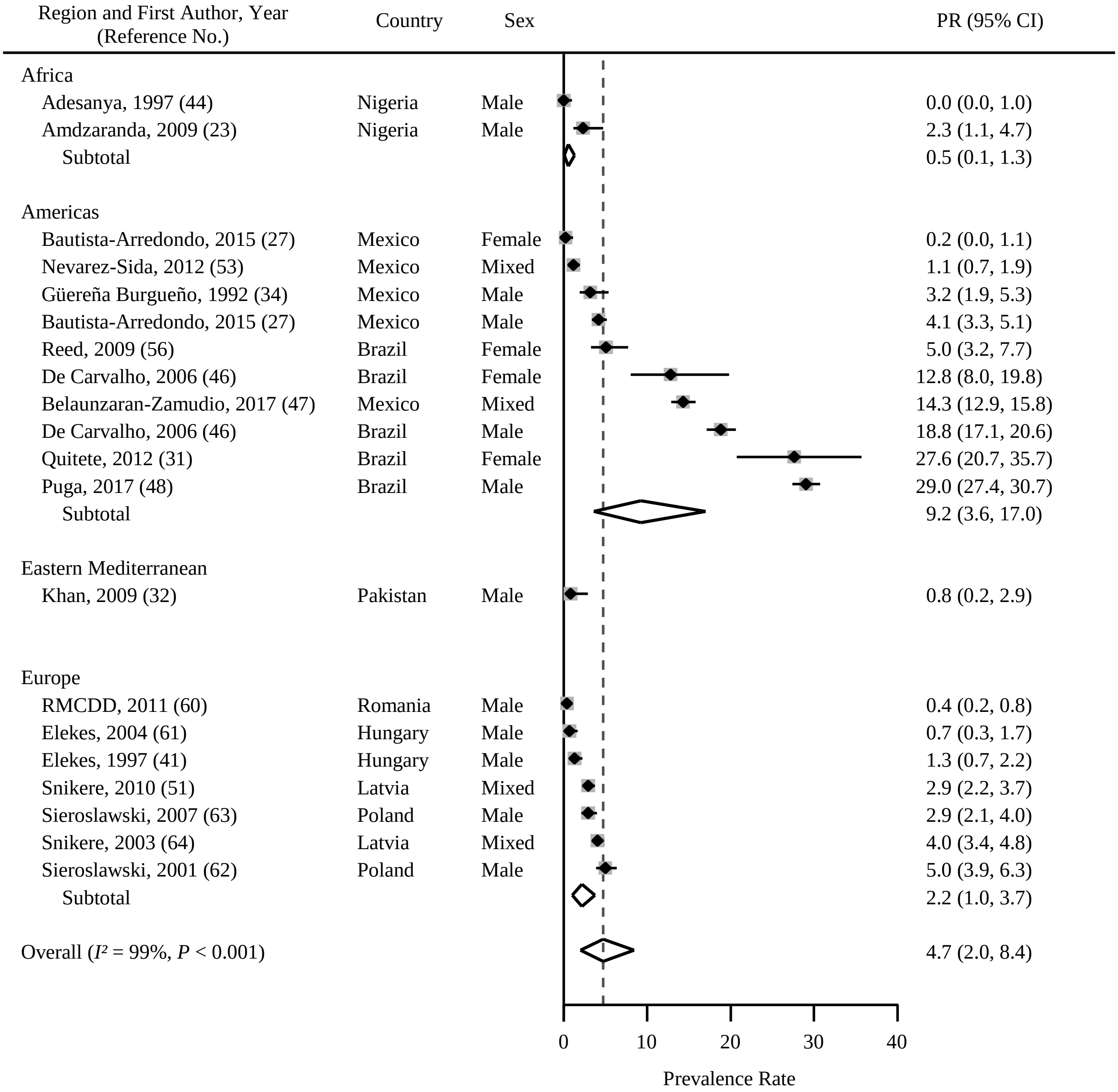

Web Figure 4. Prevalence rates and random effects meta-analyses of cocaine use during imprisonment in low- and middle-income countries by regions as defined by the World Health Organization, 1987-2017. The dashed line indicates the overall pooled random effects prevalence. CI, confidence interval; PR, prevalence rate.

Web Table 7. Meta-regression analyses of study characteristics and the prevalence of cocaine use among people who are imprisoned in low- and middle-income countries.

**Substance**	**Cocaine use**							
**Prevalence period**	***Before imprisonment***				***During imprisonment***			
	Coefficient (ß)	95% CI lower	95% CI upper	P	Coefficient (ß)	95% CI lower	95% CI upper	P
Sex	-0.230	-0.407	-0.053	**0.013**	-0.032	-0.158	0.094	0.600
Age	-0.003	-0.058	0.053	0.918	-0.009	-0.028	0.011	0.341
Sample size	-0.00003	-0.00012	0.00005	0.464	0.0000365	-7.80e-06	0.0000808	0.100
Non-response rate	0.0003	-0.005	0.006	0.898	0.0004	-0.003	0.003	0.787
Before imprisonment	n.a.				0.00016	0.00009	0.00022	<**0.001**
WHO-regions (Africa Reference category)								
Americas	0.384	0.292	0.476	<**0.001**	0.103	-0.041	0.246	0.150
Eastern Mediterranean	Dropped	-	-	-	-0.003	-0.244	0.238	0.979
Europe	0.105	0.012	0.197	**0.029**	0.014	-0.132	0.160	0.843
South East Asia	Dropped	-	-	-	Dropped			-
Western Pacific	Dropped	-	-	-	Dropped			
Multivariate meta-regression analyses retaining significant variables								
Sex	0.011	-0.088	0.112	0.811				
Americas	0.389	0.291	0.488	<**0.001**				
Europe	0.104	0.010	0.198	**0.031**				

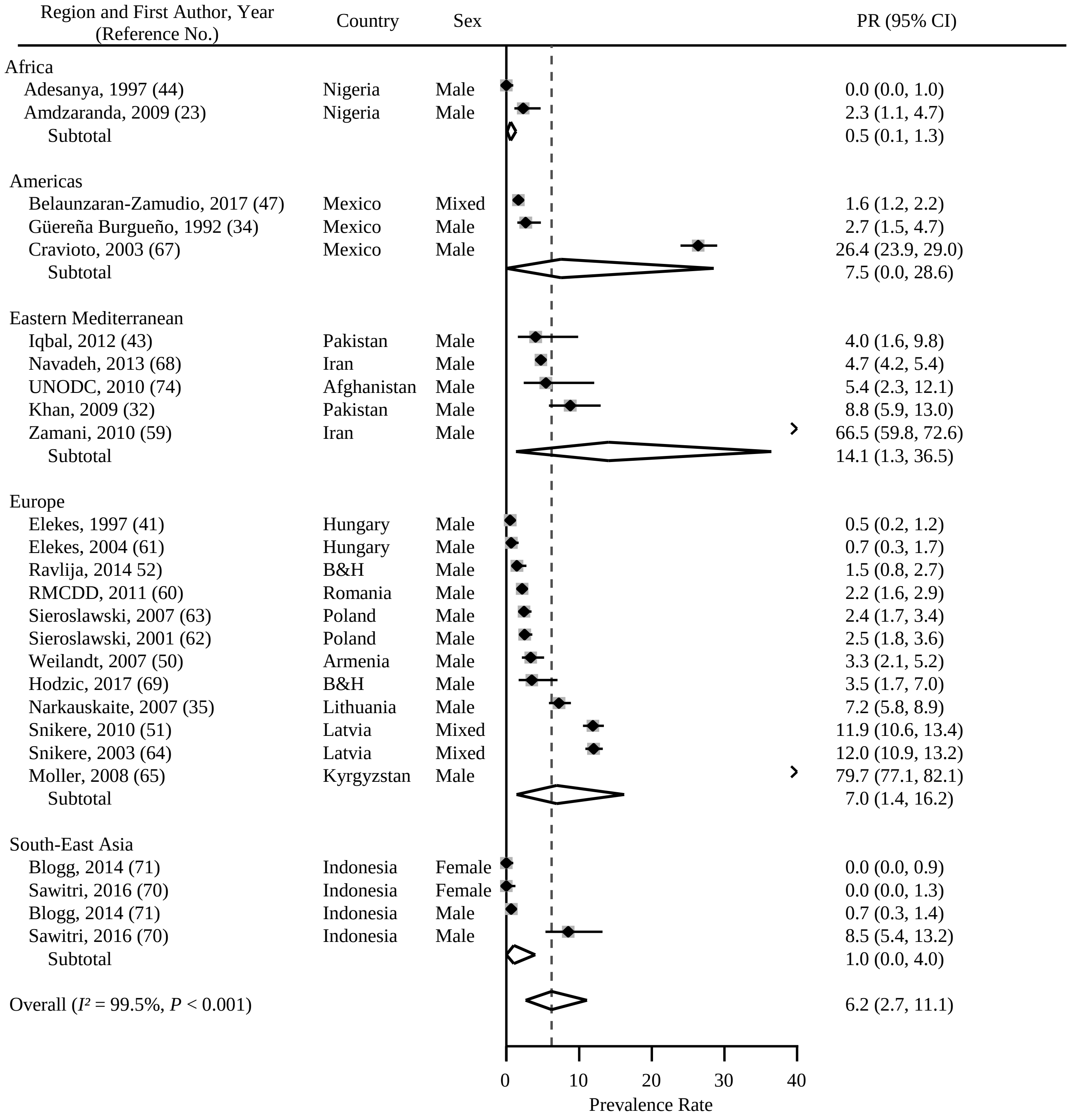

Web Figure 5. Prevalence rates and random effects meta-analyses of opiate use during imprisonment in low- and middle-income countries by regions as defined by the World Health Organization, 1987-2017. The dashed line indicates the overall pooled random effects prevalence. B&H, Bosnia and Herzegovina; CI, confidence interval; PR, prevalence rate.

Web Table 8. Meta-regression analyses of study characteristics and the prevalence of opiate use among people who are imprisoned in low- and middle-income countries.

**Substance**	**Opiate use**							
**Prevalence period**	***Before imprisonment***				***During imprisonment***			
	Coefficient (ß)	95% CI lower	95% CI upper	P	Coefficient (ß)	95% CI lower	95% CI upper	P
Sex	0.019	-0.099	0.138	0.743	0.111	-0.198	0.421	0.466
Age	0.006	-0.007	0.019	0.349	0.006	-0.032	0.043	0.759
Sample size	6.29e-06	-0.00003	0.00004	0.711	-8.02e-06	-0.0000872	0.0000712	0.836
Non-response rate	0.003	0.0009	0.005	**0.005**	-0.0008	-0.004	0.003	0.654
Before imprisonment	n.a.				0.0001843	0.0001167	0.000252	**<0.001**
WHO-regions (Africa Reference category)								
Americas	-0.0009	-0.139	0.137	0.989	0.092	-0.297	0.480	0.629
Eastern Mediterranean	0.178	0.054	0.301	**0.006**	0.170	-0.192	0.532	0.341
Europe	0.133	0.021	0.246	**0.022**	0.096	-0.231	0.422	0.550
South East Asia	0.020	-0.117	0.157	0.769	0.011	-0.361	0.382	0.953
Western Pacific	Dropped	-	-	-	Dropped	-	-	-
Multivariate meta-regression analyses retaining significant variables								
Non-response rate	0.0021	0.0005	0.0039	**0.013**				
Eastern Mediterranean	0.135	0.002	0.267	**0.046**				
Europe	0.115	-0.005	0.235	0.051				

Web Table 9. Meta-regression analyses of study characteristics and the prevalence of injection drug use among people who are imprisoned in low- and middle-income countries.

Substance	Injection drug use							
**Prevalence period**	***Before imprisonment***				***During imprisonment***			
	Coefficient (ß)	95% CI lower	95% CI upper	P	Coefficient (ß)	95% CI lower	95% CI upper	P
Sex	0.002	-0.084	0.088	0.959	0.016	-0.045	0.078	0.580
Age	0.009	-0.001	0.018	0.075	0.003	-0.005	0.011	0.376
Sample size	-6.19e-06	-0.00002	9.87e-06	0.443	-1.24e-06	-0.000017	0.00002	0.892
Non-response rate	0.002	-0.004	0.003	0.108	0.0006	-0.0007	0.002	0.337
Before imprisonment	n.a.				0.00007	-0.00005	0.00020	0.224
WHO-regions (Africa Reference category)								
Americas	0.094	-0.002	0.191	**0.054**	0.012	-0.060	0.085	0.727
Eastern Mediterranean	0.167	0.061	0.277	**0.003**	0.014	-0.069	0.100	0.727
Europe	0.195	0.093	0.297	**<0.001**	0.087	0.010	0.163	**0.028**
South East Asia	0.039	-0.087	0.167	0.533	0.013	-0.074	0.101	0.755
Western Pacific	0.013	-0.079	0.089	0.895	Dropped	-	-	-

Web Appendix 2. Nicotine use before imprisonment

Nicotine use before imprisonment was reported for 21 samples from 14 LMIC (24, 25, 27, 29, 33, 40-42, 61, 63, 78, 80, 82-87). The prevalence of nicotine use before imprisonment ranged from 33% to 93% (Web Figure 6). The heterogeneity between the studies was high (I^2 = 98.5%; P<0.001). Pooled rates indicate a prevalence of 70% (95% CI: 64, 76) for people in LMIC prisons. Nicotine use before imprisonment was significantly more frequent in imprisoned men (ß = 0.21; P = 0.04) as compared to women and in Europe (82%; ß = 0.278; P = 0.009) as compared to Africa (Web Table 3).

Web Appendix 3. Alcohol use before imprisonment

The prevalence rates of alcohol use before imprisonment were reported from 23 samples in 14 LMIC (24, 29, 33, 41, 44-47, 50, 51, 55, 61, 72, 75, 77, 78, 80-84). The prevalence of alcohol use before imprisonment ranged from 33% in Iran to 94% in Hungary (I^2 = 99.2%; P<0.001). The pooled prevalence of alcohol use before imprisonment was 71% (95% CI: 63, 78) for people imprisoned in LMIC (Web Figure 7). There were significant differences in the prevalence of alcohol use with respect to WHO-regions: lower rates in the Eastern Mediterranean (37%; ß = -0.278; *P* = 0.009) and higher rates in Europe (85%; ß = 0.151; *P* = 0.05) as compared to Africa (Web Table 4).

Web Appendix 4. Illicit drug use before imprisonment

There were 50 samples from 21 LMIC reporting prevalence estimates for illicit drug use before imprisonment (26, 27, 29, 34, 35, 38, 41, 42, 45, 47, 49, 51, 52, 54, 55, 58-61, 63-65, 67, 69, 71, 72, 77, 78, 80, 82, 84-98). Estimates for illicit drug use during imprisonment ranged from 9% for men imprisoned in China to 93% for men imprisoned in Iran (Web Figure 8). The heterogeneity between the studies was high (I^2 = 99.6%; *P*<0.001). Pooled rates of illicit drug use during imprisonment indicate a prevalence of 48% (95% CI: 41, 55) in LMIC. Meta-regression analyses did not show any significant association between study characteristics and illicit drug use before imprisonment (Web Table 5).

Web Appendix 5. Cannabis use before imprisonment

Prevalence estimates of cannabis use before imprisonment were reported for 24 samples in 12 LMIC (24, 27, 29, 40, 41, 44, 46, 47, 49-51, 55, 60- 64, 72, 77, 86, 99). Prevalence estimates for cannabis use before imprisonment ranged from 9% to 69% (Web Figure 9). The heterogeneity between the studies was high (I^2 = 99.6%; *P*<0.001). Pooled rates indicate a prevalence of 37% (95% CI: 28, 47) for cannabis use before imprisonment in LMIC. Study characteristics did not associate with cannabis use before imprisonment (Web Table 6).

Web Appendix 6. Cocaine use before imprisonment

Prevalence estimates of cocaine use during imprisonment were reported for 24 samples in 10 LMIC (24, 27, 40, 41, 44, 46, 47, 49, 51, 54, 55, 60-64, 72, 77, 86, 99, 100). Prevalence estimates for cocaine use during imprisonment ranged from 1% to 52% (Web Figure 10). The heterogeneity between the studies was high (I^2 = 99.4%; *P*<0.001). Pooled rates indicate that 23% (95% CI: 16, 30) of the people used cocaine before imprisonment in LMIC. Imprisoned women had higher rates cocaine use before imprisonment than imprisoned men (ß = -0.230, *P* = 0.01). The prevalence of cocaine use was significantly higher in the Americas (41.8%, ß = 0.345, *P* < 0.001) and in Europe (22.9%, ß = 0.105, *P* = 0.03) as compared to Africa (Web Table 7).

Web Appendix 7. Opiate use before imprisonment

Prevalence estimates of opiate use before imprisonment were reported for 37 samples in 22 LMIC (24, 26, 27, 29, 38, 40, 41, 44, 47, 49-52, 58, 60-64, 69, 71, 72, 76, 82, 83, 87, 94-96, 101-103). Prevalence estimates for opiate use before imprisonment ranged from 1% to 39% (Web Figure 11). The heterogeneity between the studies was high (I^2 = 99.3%; *P*<0.001). Pooled rates indicate a prevalence of 10% (95% CI: 3, 11) for opiate use before imprisonment in LMIC. The opiate use before imprisonment was positively associated with the non-response rate (ß = 0.003, *P* = 0.005). Rates of opiate use before imprisonment were significantly higher in the Eastern Mediterranean Region (18.1%, ß = 0.178, *P* = 0.006) and in Europe (15.5%, ß = 0.133, *P* = 0.02) (Web Table 8).

Web Appendix 8. Injection drug use before imprisonment

Estimates for injection drug use before imprisonment were reported for 55 samples from 27 LMIC (26, 27, 29, 32, 35, 38, 40, 41, 44-48, 50, 52, 54, 57, 59, 63, 66, 68-77, 81-83, 85, 87, 89, 91, 93-97, 99-106). Estimates for injection drug use before imprisonment ranged from 0% to 49% (Web Figure 12). The heterogeneity between the studies was high (I^2 = 99.3%; *P*<0.001). Pooled rates indicated a prevalence of

10% (95% CI: 7, 13) for injection drug use before imprisonment in LMIC. Injection drug use was significantly more frequent in the Americas (9.2%, ß = 0.094, *P* = 0.05), in the Eastern Mediterranean Region (15.7%, ß = 0.167, *P* = 0.003) and in Europe (18.5%, ß = 0.195, *P* = <0.001) as compared to Africa (Web Table 9).

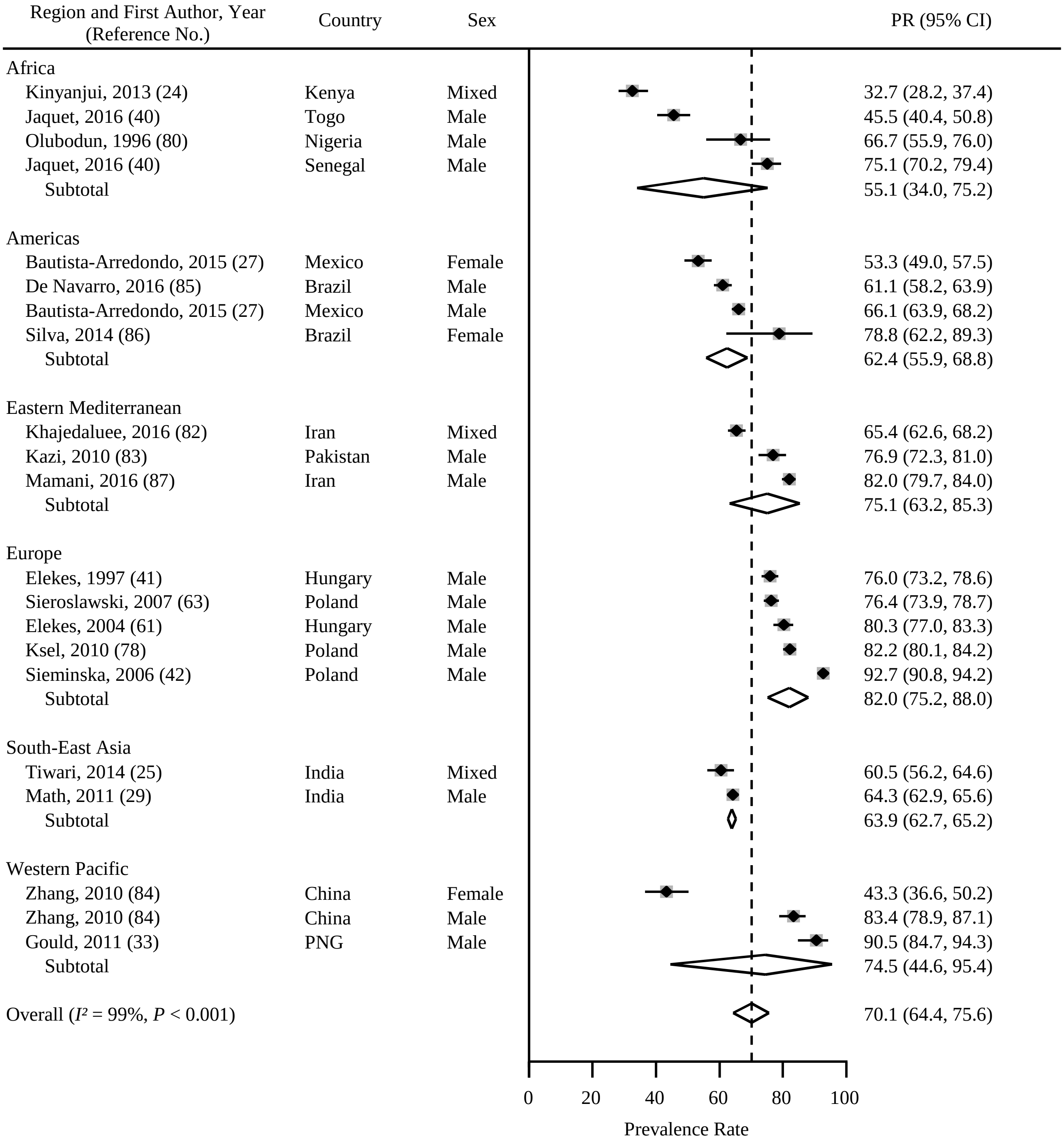

Web Figure 6. Prevalence of nicotine use before imprisonment in low- and middle-income countries by regions as defined by the World Health Organization, 1987-2017. The dashed line indicates the overall pooled random effects prevalence. CI, confidence interval; PGN, Papua New Guinea; PR, prevalence rate.

Region and First Author, Year (Reference No.)	Country	Sex	PR (95% CI)
Africa			
Adoga, 2009 (75)	Nigeria	Male	60.0 (54.4, 65.4)
Akeke, 2009 (45)	Leshoto	Male	65.0 (56.3, 72.9)
Kinyanjui, 2013 (24)	Kenya	Mixed	65.1 (60.2, 69.6)
Olubodun, 1996 (80)	Nigeria	Male	77.8 (67.6, 85.5)
Adesanya, 1997 (44)	Nigeria	Male	80.3 (76.0, 83.9)
Subtotal			69.8 (60.7, 78.1)
Americas			
Miranda, 2000 (77)	Brazil	Female	48.8 (40.0, 57.6)
Canazaro, 2010 (55)	Brazil	Female	53.7 (47.9, 59.3)
Falquetto, 2013 (81)	Brazil	Mixed	68.8 (65.3, 72.0)
De Carvalho, 2006 (46)	Brazil	Female	69.6 (61.1, 77.0)
Santos, 2011 (72)	Brazil	Mixed	73.0 (68.6, 77.0)
De Carvalho, 2006 (46)	Brazil	Male	74.1 (72.1, 76.0)
Belaunzaran-Zamudio, 2017 (47)	Mexico	Mixed	78.3 (76.5, 79.9)
Subtotal			67.6 (61.4, 73.5)
Eastern Mediterranean			
Khajedaluee, 2016 (82)	Iran	Mixed	32.6 (29.9, 35.4)
Kazi, 2010 (83)	Pakistan	Male	51.9 (46.8, 57.0)
Subtotal			37.2 (34.8, 39.7)
Europe			
Weilandt, 2007 (50)	Armenia	Male	65.7 (61.6, 69.6)
Ksel, 2010 (78)	Poland	Male	81.5 (79.3, 83.5)
Snikere, 2010 (51)	Latvia	Mixed	87.0 (85.5, 88.4)
Elekes, 1997 (41)	Hungary	Male	92.6 (90.8, 94.1)
Elekes, 2004 (61)	Hungary	Male	93.8 (91.6, 95.4)
Subtotal			85.3 (77.1, 92.0)
South-East Asia			
Math, 2011 (29)	India	Male	51.4 (50.0, 52.8)
Western Pacific			
Zhang, 2010 (84)	China	Female	59.2 (52.3, 65.8)
Zhang, 2010 (84)	China	Male	81.8 (77.1, 85.7)
Gould, 2011 (33)	PNG	Male	91.9 (86.4, 95.3)
Subtotal			79.1 (59.6, 93.3)
Overall (I^2 = 99%, P < 0.001)			71.0 (63.3, 78.2)

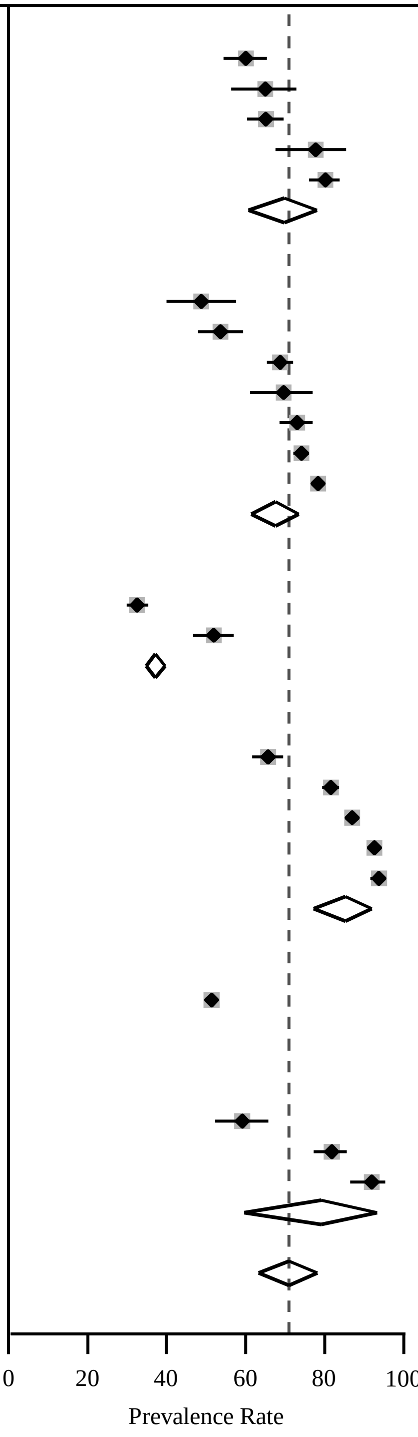

Web Figure 7. The prevalence of alcohol use before imprisonment in people who are imprisoned in low- and middle-income countries by regions as defined by the World Health Organization, 1987-2017. The dashed line indicates the overall pooled random effects prevalence. CI, confidence interval; PGN, Papua New Guinea; PR, prevalence rate.

Region and First Author, Year (Reference No.)	Country	Sex	PR (95% CI)
**Africa**			
Olubodun, 1996 (80)	Nigeria	Male	34.6 (25.1, 45.4)
Akeke, 2009 (45)	Leshoto	Male	45.5 (37.0, 54.3)
Adesokan, 2014 (88)	Nigeria	Male	56.7 (49.1, 64.1)
Subtotal			46.0 (33.9, 58.4)
**Americas**			
Bautista-Arredondo, 2015 (27)	Mexico	Female	40.6 (36.4, 44.9)
Ferreira, 1996 (89)	Brazil	Female	47.7 (42.5, 52.9)
Canazaro, 2010 (55)	Brazil	Female	54.4 (48.6, 60.0)
Miranda, 2000 (77)	Brazil	Female	54.5 (45.7, 63.1)
Santos Barros, 2013 (90)	Brazil	Female	56.8 (48.7, 64.5)
Güereña Burgueño, 1992 (34)	Mexico	Male	58.8 (54.0, 63.4)
Monsalve-Castillo, 2009 (91)	Venezuela	Male	60.0 (53.1, 66.5)
Belaunzaran-Zamudio, 2017 (47)	Mexico	Mixed	60.2 (58.2, 62.3)
Bautista-Arredondo, 2015 (27)	Mexico	Male	61.7 (59.5, 63.8)
Cravioto, 2003 (67)	Mexico	Male	69.8 (67.0, 72.3)
Santos, 2011 (72)	Brazil	Mixed	73.7 (69.3, 77.7)
De Navarro, 2016 (85)	Brazil	Male	75.0 (72.4, 77.4)
Silva, 2014 (86)	Brazil	Female	75.8 (59.0, 87.2)
Marins, 2000 (92)	Brazil	Male	78.2 (75.4, 80.8)
El Maerrawi, 2015 (54)	Brazil	Male	80.4 (76.9, 83.5)
Coelho, 2009 (93)	Brazil	Male	90.1 (86.4, 92.9)
Subtotal			65.4 (59.4, 71.2)
**Eastern Mediterranean**			
Nokhodian, 2012 (94)	Iran	Female	30.1 (23.5, 37.5)
UNODC, 2010 (74)	Afghanistan	Male	54.3 (44.2, 64.1)
Khajedaluee, 2016 (82)	Iran	Mixed	54.4 (51.5, 57.3)
Ziaee, 2014 (95)	Iran	Mixed	64.5 (61.3, 67.6)
Mamani, 2016 (87)	Iran	Male	75.7 (73.2, 78.0)
Zamani, 2010 (59)	Iran	Male	93.1 (88.8, 95.8)
Subtotal			63.6 (50.0, 76.2)
**Europe**			
Winetsky, 2014 (26)	Tajikistan	Male	12.2 (10.6, 14.1)
Ksel, 2010 (78)	Poland	Male	13.7 (12.0, 15.7)
RMCDD, 2007 (49)	Romania	Male	18.5 (17.2, 19.9)
Sieminska, 2006 (42)	Poland	Male	20.8 (18.3, 23.6)
Burek, 2010 (96)	Croatia	Female	21.8 (16.5, 28.2)
Elekes, 1997 (61)	Hungary	Male	22.1 (19.6, 24.8)
RMCDD, 2011 (60)	Romania	Male	25.6 (23.7, 27.5)
Burek, 2010 (96)	Croatia	Male	26.2 (24.7, 27.8)
Narkauskaite, 2007 (35)	Lithuania	Female	31.3 (21.5, 43.2)
Elekes, 2004 (61)	Hungary	Male	31.5 (28.0, 35.3)
Azbel, 2015 (97)	Azerbaijan	Mixed	33.1 (29.2, 37.3)
Ravlija, 2014 (52)	B&H	Male	37.3 (33.5, 41.1)
Sieroslawski, 2007 (63)	Poland	Male	48.9 (46.1, 51.7)
Narkauskaite, 2007 (35)	Lithuania	Male	49.9 (47.0, 52.8)
Snikere, 2003 (64)	Latvia	Mixed	53.0 (51.2, 54.8)
Hodzic, 2017 (69)	B&H	Male	53.5 (46.6, 60.3)
Narkauskaite, 2010 (35)	Lithuania	Female	57.7 (46.2, 68.5)
Snikere, 2010 (51)	Latvia	Mixed	61.1 (58.9, 63.3)
Moller, 2008 (65)	Kyrgyzstan	Male	85.6 (83.2, 87.6)
Subtotal			36.3 (27.2, 45.9)
**South-East Asia**			
Math, 2011 (29)	India	Male	13.0 (12.1, 13.9)
Blogg, 2014 (71)	Indonesia	Male	37.2 (34.1, 40.4)
Blogg, 2014 (71)	Indonesia	Female	41.3 (36.6, 46.2)
Subtotal			29.4 (11.0, 52.3)
**Western Pacific**			
Zhang, 2010 (84)	China	Male	9.3 (6.5, 13.0)
Zhang, 2010 (84)	China	Female	21.4 (16.3, 27.6)
Zhu, 2017 (98)	China	Female	25.9 (24.3, 27.6)
Subtotal			18.3 (8.7, 30.5)
Overall ($I^2 = 99.6\%$, $P < 0.001$)			47.7 (40.8, 54.8)

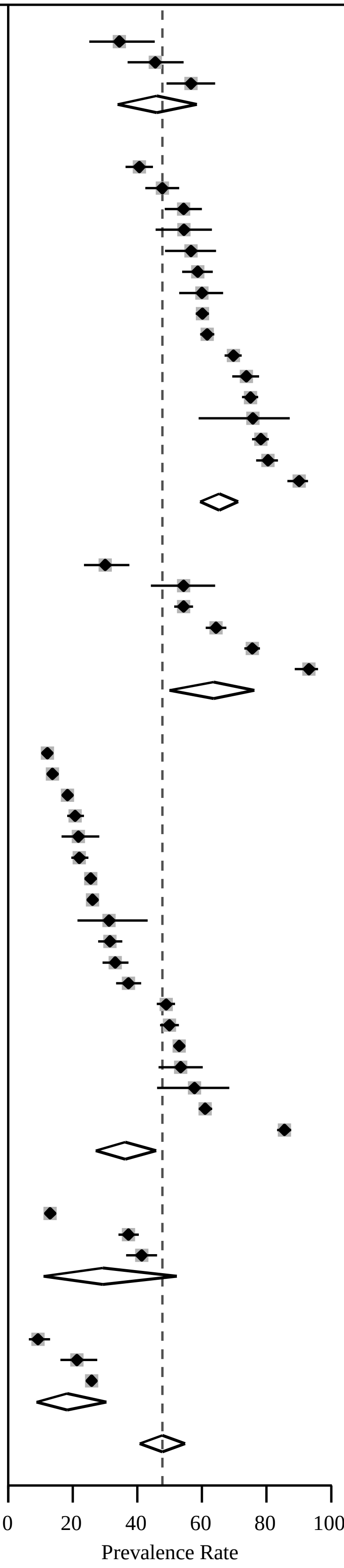

Web Figure 8. The prevalence of illicit drug use before imprisonment in low- and middle-income countries by regions as defined by the World Health Organization, 1987-2017. The dashed line indicates the overall pooled random effects prevalence. B&H, Bosnia and Herzegovina; CI, confidence interval; PR, prevalence rate.

Region and First Author, Year (Reference No.)	Country	Sex	PR (95% CI)
Africa			
Kinyanjui, 2013 (24)	Kenya	Mixed	21.0 (17.3, 25.3)
Jaquet, 2016 (40)	Togo	Male	22.2 (18.1, 26.9)
Adesanya, 1997 (44)	Nigeria	Male	33.9 (29.4, 38.7)
Jaquet, 2016 (40)	Senegal	Male	45.9 (40.7, 51.3)
Subtotal			30.3 (19.9, 41.9)
Americas			
Bautista-Arredondo, 2015 (27)	Mexico	Female	28.1 (24.4, 32.1)
Belaunzaran-Zamudio, 2017 (47)	Mexico	Mixed	40.6 (38.6, 42.7)
De Carvalho, 2006 (46)	Brazil	Female	43.2 (34.8, 52.0)
Canazaro, 2010 (55)	Brazil	Female	47.0 (41.3, 52.8)
Miranda, 2000 (77)	Brazil	Female	47.9 (39.2, 56.8)
Bautista-Arredondo, 2015 (27)	Mexico	Male	49.1 (46.9, 51.3)
Strazza, 2004 (99)	Brazil	Female	56.2 (50.5, 61.8)
De Carvalho, 2006 (46)	Brazil	Male	61.6 (59.4, 63.8)
Silva, 2014 (86)	Brazil	Female	63.6 (46.6, 77.8)
Santos, 2011 (72)	Brazil	Mixed	68.7 (64.1, 73.0)
Subtotal			50.2 (42.5, 57.9)
Europe			
RMCDD, 2011 (60)	Romania	Male	9.4 (8.2, 10.7)
RMCDD, 2007 (49)	Romania	Male	9.6 (8.6, 10.7)
Elekes, 1997 (41)	Hungary	Male	10.7 (8.9, 12.9)
Elekes, 2004 (61)	Hungary	Male	26.3 (22.9, 29.9)
Weilandt, 2007 (50)	Armenia	Male	34.7 (30.8, 38.8)
Sieroslawski, 2001 (62)	Poland	Male	38.7 (36.0, 41.5)
Sieroslawski, 2007 (63)	Poland	Male	43.1 (40.3, 45.8)
Snikere, 2003 (64)	Latvia	Mixed	51.0 (49.2, 52.8)
Snikere, 2010 (51)	Latvia	Mixed	60.6 (58.4, 62.7)
Subtotal			29.9 (16.2, 45.7)
South-East Asia			
Math, 2011 (29)	India	Male	12.2 (11.4, 13.2)
Overall (I^2 = 99.6%, P < 0.001)			37.3 (28.4, 46.6)

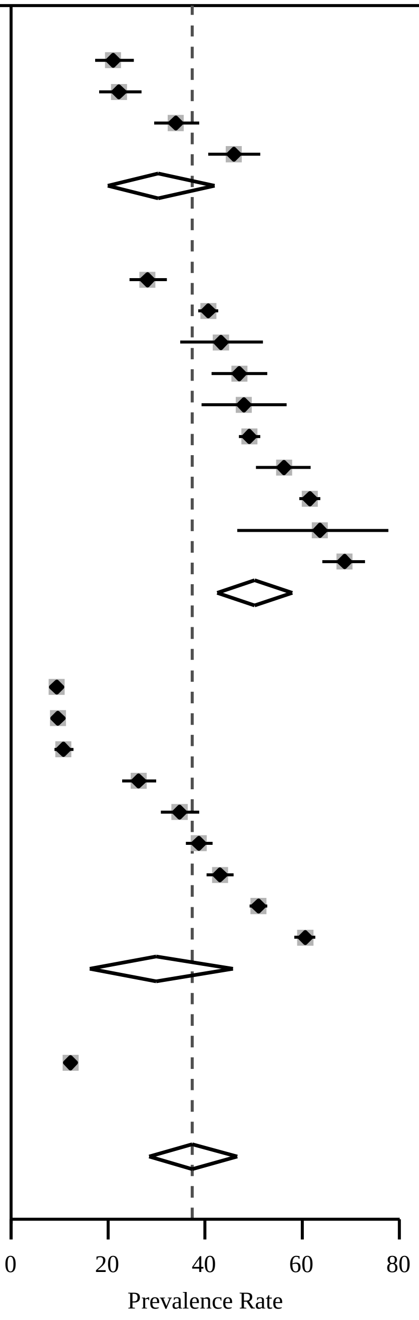

Web Figure 9. Random effects meta-analyses for the prevalence of cannabis use before imprisonment in low- and middle-income countries by regions as defined by the World Health Organization, 1987-2017. The dashed line indicates the overall pooled random effects prevalence. CI, confidence interval; PR, prevalence rate.

Region and First Author, Year (Reference No.)	Country	Sex	PR (95% CI)
Africa			
Jaquet, 2016 (40)	Togo	Male	0.9 (0.3, 2.5)
Jaquet, 2016 (40)	Senegal	Male	1.2 (0.5, 3.0)
Kinyanjui, 2013 (24)	Kenya	Mixed	2.3 (1.2, 4.3)
Adesanya, 1997 (44)	Nigeria	Male	6.6 (4.5, 9.5)
Subtotal			2.4 (0.6, 5.1)
Americas			
Bautista-Arredondo, 2015 (27)	Mexico	Female	26.2 (22.6, 30.1)
De Albuquerque, 2014 (100)	Brazil	Male	34.1 (31.3, 36.9)
El Maerrawi, 2015 (54)	Brazil	Male	37.4 (33.4, 41.5)
Canazaro, 2010 (55)	Brazil	Female	38.3 (32.9, 44.1)
Bautista-Arredondo, 2015 (27)	Mexico	Male	38.7 (36.6, 40.9)
Belaunzaran-Zamudio, 2017 (47)	Mexico	Mixed	41.1 (39.1, 43.2)
Strazza, 2004 (99)	Brazil	Female	44.8 (39.2, 50.6)
De Carvalho, 2006 (46)	Brazil	Female	47.2 (38.7, 55.9)
Santos, 2011 (72)	Brazil	Mixed	48.6 (43.8, 53.3)
Miranda, 2000 (77)	Brazil	Female	51.2 (42.4, 60.0)
Silva, 2014 (86)	Brazil	Female	51.5 (35.2, 67.5)
De Carvalho, 2006 (46)	Brazil	Male	52.0 (49.8, 54.3)
Subtotal			41.8 (37.2, 46.4)
Europe			
Elekes, 1997 (41)	Hungary	Male	5.2 (3.9, 6.7)
RMCDD, 2007 (49)	Romania	Male	6.2 (5.4, 7.1)
RMCDD, 2011 (60)	Romania	Male	9.3 (8.1, 10.6)
Elekes, 2004 (61)	Hungary	Male	11.2 (8.9, 13.9)
Snikere, 2003 (64)	Latvia	Mixed	15.0 (13.7, 16.4)
Snikere, 2010 (51)	Latvia	Mixed	19.0 (17.3, 20.8)
Sieroslawski, 2001 (62)	Poland	Male	19.3 (17.1, 21.6)
Sieroslawski, 2007 (63)	Poland	Male	20.8 (18.6, 23.2)
Subtotal			12.6 (8.6, 17.3)
Overall (I^2 = 99%, P < 0.001)			22.9 (16.3, 30.2)

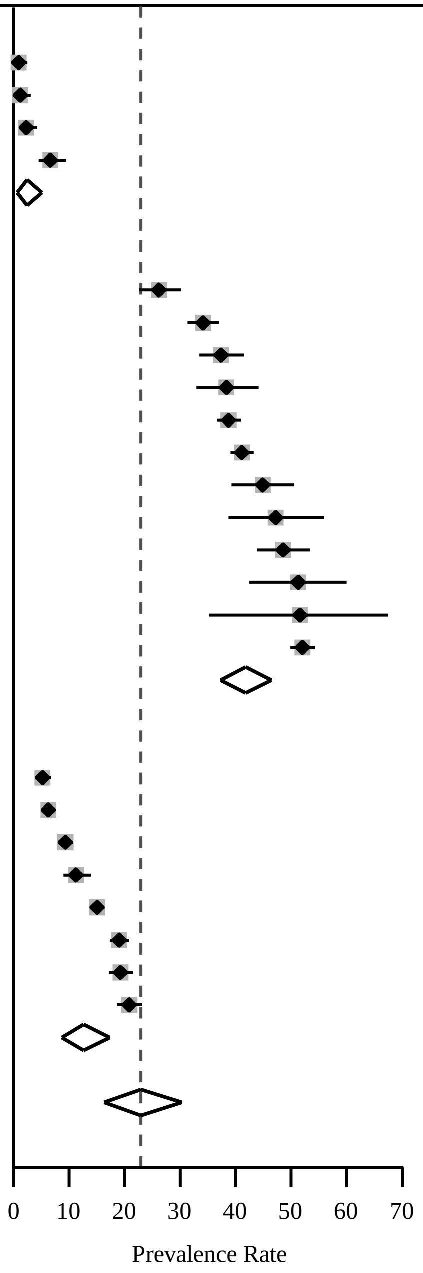

Web Figure 10. Random effects meta-analyses for the prevalence of cocaine use before imprisonment in low- and middle-income countries by WHO-Region, 1987-2017. The dashed line indicates the overall pooled random effects prevalence. CI, confidence interval; PR, prevalence rate.

Region and First Author, Year (Reference No.)	Country	Sex	PR (95% CI)
**Africa**			
Jaquet, 2016 (40)	Togo	Male	0.6 (0.2, 2.1)
Jaquet, 2016 (40)	Senegal	Male	0.6 (0.2, 2.2)
Kinyanjui, 2013 (24)	Kenya	Mixed	1.3 (0.5, 2.9)
Adesanya, 1997 (44)	Nigeria	Male	7.8 (5.6, 10.9)
Subtotal			1.9 (0.1, 5.5)
**Americas**			
Santos, 2011 (72)	Brazil	Mixed	0.5 (0.1, 1.7)
Bautista-Arredondo, 2015 (27)	Mexico	Female	1.2 (0.5, 2.5)
Bautista-Arredondo, 2015 (27)	Mexico	Male	2.2 (1.6, 2.9)
Belaunzaran-Zamudio, 2017 (47)	Mexico	Mixed	5.6 (4.8, 6.7)
Subtotal			2.1 (0.5, 4.6)
**Eastern Mediterranean**			
Nokhodian, 2012 (94)	Iran	Female	3.1 (1.3, 7.0)
Mohamed, 2013 (101)	Egypt	Mixed	8.4 (6.3, 11.2)
Khajedaluee, 2016 (82)	Iran	Mixed	10.0 (8.3, 11.9)
Kazi, 2010 (83)	Pakistan	Male	13.7 (10.6, 17.7)
Mamani, 2016 (87)	Iran	Male	17.1 (15.0, 19.3)
UNODC, 2010 (74)	Afghanistan	Male	30.4 (22.0, 40.5)
Ziaee, 2014 (95)	Iran	Mixed	36.8 (33.7, 40.0)
Memon, 2012 (102)	Pakistan	Male	39.0 (37.1, 41.1)
Subtotal			18.1 (9.4, 28.9)
**Europe**			
Elekes, 1997 (41)	Hungary	Male	3.0 (2.1, 4.3)
Winetsky, 2014 (26)	Tajikistan	Male	4.2 (3.2, 5.4)
Elekes, 2004 (61)	Hungary	Male	5.7 (4.2, 7.9)
Sieroslawski, 2007 (63)	Poland	Male	8.4 (7.0, 10.1)
RMCDD, 2007 (49)	Romania	Male	8.4 (7.5, 9.4)
Sieroslawski, 2001 (62)	Poland	Male	9.5 (8.0, 11.3)
RMCDD, 2011 (59)	Romania	Male	11.7 (10.4, 13.1)
Weilandt, 2007 (50)	Armenia	Male	15.7 (12.9, 19.0)
Ravlija, 2014 (52)	B&H	Male	17.3 (14.5, 20.4)
Burek, 2010 (96)	Croatia	Female	19.1 (14.2, 25.4)
Snikere, 2003 (64)	Latvia	Mixed	24.0 (22.5, 25.6)
Burek, 2010 (96)	Croatia	Male	24.7 (23.2, 26.2)
Hodzic, 2017 (69)	B&H	Male	28.5 (22.7, 35.1)
Azbel, 2016 (103)	Kyrgyzstan	Mixed	28.8 (24.4, 33.6)
Snikere, 2010 (51)	Latvia	Mixed	29.4 (27.4, 31.5)
Narkauskaite, 2010 (38)	Lithuania	Female	33.8 (23.9, 45.4)
Subtotal			15.5 (10.9, 20.6)
**South-East Asia**			
Math, 2011 (29)	India	Male	0.6 (0.4, 0.9)
Niriella, 2015 (104)	Sri Lanka	Female	2.9 (0.8, 10.1)
Niriella, 2015 (104)	Sri Lanka	Male	4.6 (2.8, 7.5)
Blogg, 2014 (71)	Indonesia	Female	6.2 (4.2, 9.0)
Blogg, 2014 (71)	Indonesia	Male	8.3 (6.7, 10.3)
Subtotal			4.0 (0.6, 9.9)
Overall (I^2 = 99%, P < 0.001)			10.4 (7.0, 14.4)

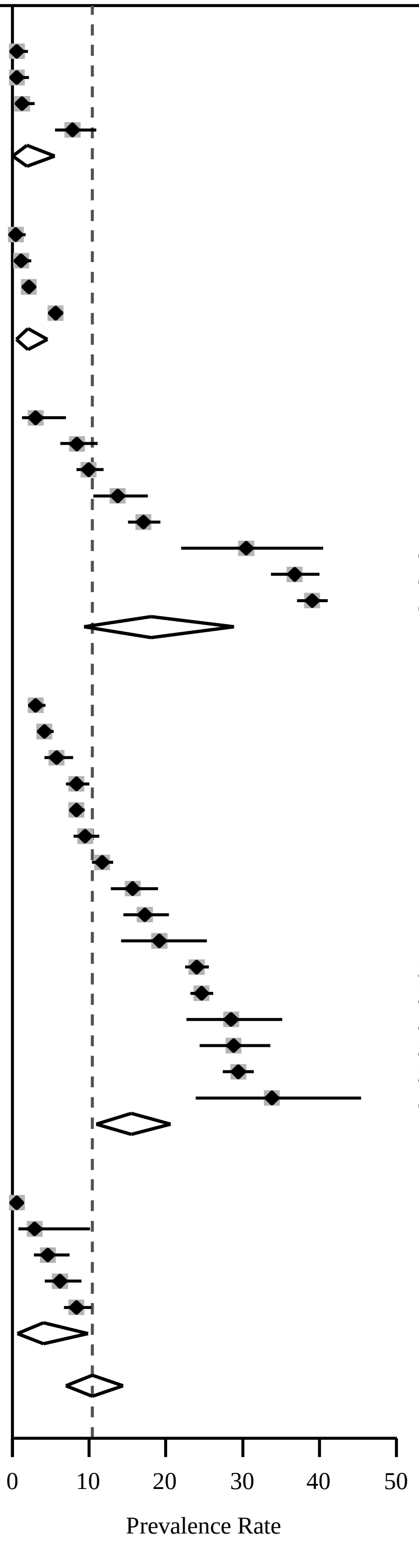

Web Figure 11. Random effects meta-analyses for the prevalence of opiate use before imprisonment in low- and middle-income countries by regions as defined by the World Health Organization, 1987-2017. The dashed line indicates the overall pooled random effects prevalence. B&H, Bosnia and Herzegovina; CI, confidence interval; PR, prevalence rate.

Region and First Author, Year (Reference No.)	Country	Sex	PR (95% CI)
**Africa**			
Vaz, 1995 (66)	Mozambique	Male	0.0 (0.0, 0.3)
Vaz, 1995 (66)	Mozambique	Female	0.0 (0.0, 6.6)
Adesanya, 1997 (44)	Nigeria	Male	0.0 (0.0, 1.0)
Adoga, 2009 (75)	Nigeria	Male	0.0 (0.0, 1.3)
Jaquet, 2016 (40)	Togo	Male	0.6 (0.2, 2.1)
Jaquet, 2016 (40)	Senegal	Male	0.6 (0.2, 2.2)
Akeke, 2009 (45)	Leshoto	Male	3.3 (1.3, 8.1)
Subtotal			0.2 (0.0, 0.8)
**Americas**			
De Navarro, 2016 (85)	Brazil	Male	2.7 (1.9, 3.8)
De Albuquerque, 2014 (100)	Brazil	Male	3.3 (2.4, 4.5)
Bautista-Arredondo, 2015 (27)	Mexico	Female	3.5 (2.2, 5.4)
Santos Barros, 2013 (90)	Brazil	Female	4.1 (1.9, 8.6)
De Carvalho, 2006 (46)	Brazil	Male	5.0 (4.1, 6.1)
Bautista-Arredondo, 2015 (27)	Mexico	Male	5.3 (4.4, 6.4)
El Maerrawi, 2015 (54)	Brazil	Male	5.3 (3.7, 7.5)
Monsalve-Castillo, 2009 (91)	Venezuela	Male	6.0 (3.5, 10.2)
Falquetto, 2013 (81)	Brazil	Mixed	6.2 (4.6, 8.1)
Strazza, 2004 (99)	Brazil	Female	8.3 (5.6, 12.0)
Coelho, 2009 (93)	Brazil	Male	8.7 (6.1, 12.2)
Belaunzaran-Zamudio, 2017 (47)	Mexico	Mixed	9.2 (8.1, 10.5)
Santos, 2011 (72)	Brazil	Mixed	10.2 (7.7, 13.4)
De Carvalho, 2006 (46)	Brazil	Female	10.4 (6.2, 17.0)
Miranda, 2000 (77)	Brazil	Female	10.7 (6.4, 17.5)
Macri Troya, 2010 (73)	Uruguay	Male	15.1 (11.5, 19.7)
Güereña Burgueño, 1992 (34)	Mexico	Male	20.7 (17.1, 24.9)
Massad, 1999 (105)	Brazil	Male	21.6 (18.5, 24.9)
Marins, 2000 (92)	Brazil	Male	22.2 (19.6, 25.0)
Ferreira, 1996 (89)	Brazil	Female	22.9 (18.8, 27.5)
Subtotal			9.2 (6.6, 12.1)
**Eastern Mediterranean**			
Nokhodian, 2012 (94)	Iran	Female	3.1 (1.3, 7.0)
Ziaee, 2014 (95)	Iran	Mixed	6.7 (5.2, 8.5)
Mohamed, 2013 (101)	Egypt	Mixed	8.4 (6.3, 11.2)
Khajedaluee, 2016 (82)	Iran	Mixed	10.0 (8.3, 11.9)
Kazi, 2010 (83)	Pakistan	Male	13.7 (10.6, 17.7)
Navadeh, 2013 (68)	Iran	Male	16.0 (15.0, 17.1)
Mamani, 2016 (87)	Iran	Male	17.1 (15.0, 19.3)
Memon, 2012 (102)	Pakistan	Male	39.0 (37.1, 41.1)
Zamani, 2010 (59)	Iran	Male	42.4 (35.8, 49.2)
Subtotal			15.7 (8.9, 23.8)
**Europe**			
Sieroslawski, 2007 (63)	Poland	Male	0.7 (0.3, 1.3)
Elekes, 1997 (41)	Hungary	Male	1.2 (0.6, 2.1)
Winetsky, 2014 (26)	Tajikistan	Male	4.2 (3.2, 5.4)
Weilandt, 2007 (50)	Armenia	Male	13.3 (10.7, 16.4)
Ravlija, 2014 (52)	B&H	Male	17.3 (14.5, 20.4)
Burek, 2010 (96)	Croatia	Female	19.1 (14.2, 25.4)
Snikere, 2003 (64)	Latvia	Mixed	24.0 (22.5, 25.6)
Burek, 2010 (96)	Croatia	Male	24.7 (23.2, 26.2)
Hodzic, 2017 (69)	B&H	Male	28.5 (22.7, 35.1)
Azbel, 2016 (76)	Kyrgyzstan	Mixed	29.6 (25.2, 34.5)
Azbel, 2015 (97)	Azerbaijan	Mixed	31.6 (27.7, 35.7)
Narkauskaite, 2010 (38)	Lithuania	Female	33.8 (23.9, 45.4)
Azbel, 2013 (103)	Ukraine	Mixed	48.8 (43.9, 53.6)
Subtotal			18.5 (10.8, 27.9)
**South-East Asia**			
Math, 2011 (29)	India	Male	0.2 (0.1, 0.3)
Niriella, 2015 (104)	Sri Lanka	Female	2.9 (0.8, 10.1)
Niriella, 2015 (104)	Sri Lanka	Male	4.6 (2.8, 7.5)
Blogg, 2014 (71)	Indonesia	Female	6.2 (4.2, 9.0)
Blogg, 2014 (71)	Indonesia	Male	8.3 (6.7, 10.3)
Subtotal			3.8 (0.2, 10.6)
**Western Pacific**			
Zhang, 2012 (106)	China	Male	1.9 (1.6, 2.1)
Overall (I^2 = 99%, P < 0.001)			9.5 (6.7, 12.7)

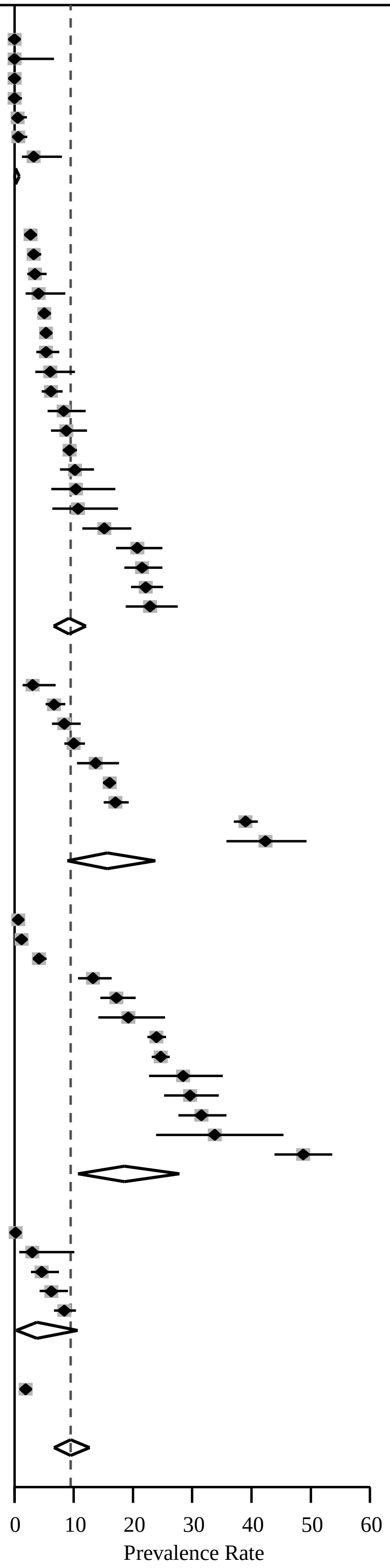

Web Figure 12: Random effects meta-analyses for the prevalence of injection drug use before imprisonment in low- and middle-income countries by regions as defined by the World Health Organization, 1987-2017. The dashed line indicates the overall pooled random effects prevalence. B&H, Bosnia and Herzegovina; CI, confidence interval; PR, prevalence rate.

Web Table 10. Samples reporting the prevalence of substance use for people imprisoned in low- and middle-income countries, 1987-2017. N.r., not reported.

First Author, Year (Reference No.)	Year of data collection (final)	Sex	Region (according to the WHO)	Country	Sample size	Non-response rate (%)	Mean Age (years)
Adesanya, 1997 (44)	1995	Male	Africa	Nigeria	395	4.8	30.4
Adesokan, 2014 (88)	2013	Male	Africa	Nigeria	164	n.r.	n.r.
Adoga, 2009 (75)	2007	Male	Africa	Nigeria	300	n.r.	29.2
Akeke, 2009 (45)	n.r.	Male	Africa	Leshoto	123	11.0	n.r.
Amdzaranda, 209 (23)	2006	Male	Africa	Nigeria	303	13.9	31.2
Azbel, 2013 (104)	2011	Mixed	Europe	Ukraine	402	5.6	31.9
Azbel, 2015 (97)	2014	Mixed	Europe	Azerbaijan	510	4.7	38.2
Azbel, 2016 (76)	2014	Mixed	Europe	Kyrgyzstan	368	3.4	37.4
Bautista-Arredondo, 2015 (27)	2010	Male	Americas	Mexico	1934	1.1	33.8
Bautista-Arredondo, 2015 (27)	2010	Female	Americas	Mexico	520	2.8	34.9
Belaunzaran-Zamudio, 2017 (47)	2012	Mixed	Americas	Mexico	2218	48.0	n.r.
Blogg, 2014 (71)	2010	Male	South-East Asia	Indonesia	900	6.0	32.0
Blogg, 2014 (71)	2010	Female	South-East Asia	Indonasia	402	5.0	34.0
Burek, 2010 (96)	2007	Female	Europe	Croatia	188	24.7	32.0
Burek, 2010 (96)	2007	Male	Europe	Croatia	3160	33.5	32.0
Canazaro, 2010 (55)	2008	Female	Americas	Brazil	287	22.0	33.4
Coelho, 2009 (93)	2003	Male	Americas	Brazil	333	n.r.	30.1
Cravioto, 2003 (67)	2000	Male	Americas	Mexico	1151	4.7	n.r.
Dadi, 2016 (39)	2015	Mixed	Africa	Ethiopia	649	7.3	30.6
De Albuquerque, 2014 (100)	2011	Male	Americas	Brazil	1097	0.3	28.6
De Carvalho, 2006 (46)	1998	Male	Americas	Brazil	1914	n.r.	30.1

De Carvalho, 2006 (46)	1998	Female	Americas	Brazil	125	n.r.	32.9
De Navarro, 2016 (85)	2013	Male	Americas	Brazil	1120	25.0	29.0
Diendere, 2011 (57)	2009	Male	Africa	Burkina Faso	300	n.r.	30.1
El Maerrawi, 2015 (54)	2007	Male	Americas	Brazil	546	16.0	29.8
Elekes, 1997 (41)	1997	Male	Europe	Hungary	951	4.9	n.r.
Elekes, 2004 (61)	2004	Male	Europe	Hungary	609	0.0	34.5
Falquetto, 2013 (81)	2010	Mixed	Americas	Brazil	730	18.5	n.r.
Ferreira, 1996 (89)	1993	Female	Americas	Brazil	350	7.4	29.0
Gould, 2011 (33)	2010	Male	Western Pacific	Papua New Guinea	148	41.7	31.0
Güereña Burgueño, 1992 (34)	1988	Male	Americas	Mexico	410	2.0	29.0
Hodzic, 2017 (69)	2013	Male	Europe	Bosnia and Herzegovina	200	73.9	32.3
Hussain, 2003 (28)	2001	Male	Eastern Mediterranean	Pakistan	425	0.0	34.8
Iqbal, 2012 (43)	2006	Male	Eastern Mediterranean	Pakistan	100	0.0	35.0
Jaka, 2014 (30)	2013	Mixed	Europe	Albania	401	n.r.	31.4
Jaquet, 2016 (40)	2013	Male	Africa	Togo	347	13.6	30.0
Jaquet, 2016 (40)	2014	Male	Africa	Senegal	333	13.6	30.0
Kazi, 2010 (83)	2008	Male	Eastern Mediterranean	Pakistan	364	5.2	27.0
Khajedaluee, 2016 (82)	2008	Mixed	Eastern Mediterranean	Iran	1114	5.2	35.1
Khan, 2009 (32)	2008	Male	Eastern Mediterranean	Pakistan	250	n.r.	44.0
Kinyanjui, 2013 (24)	2011	mixed	Africa	Kenya	395	n.r.	33.3
Ksel, 2010 (78)	2003	Male	Europe	Poland	1305	n.r.	n.r.
Macri Troya, 2010 (73)	2005	Male	Americas	Uruguay	291	0.0	29.6
Mamani, 2016 (87)	2013	Male	Eastern Mediterranean	Iran	1208	51.7	n.r.
Marins, 2000 (92)	1995	Male	Americas	Brazil	914	13.7	29.0

Massad, 1999 (105)	1994	Male	Americas	Brazil	631	5.5	30.8
Math, 2011 (29)	2009	Male	South-East Asia	India	5024	3.4	30.7
Memon, 2012 (102)	2008	Male	Eastern Mediterranean	Pakistan	2285	n.r.	42.5
Miranda, 2000 (77)	1997	Female	Americas	Brazil	121	0.8	30.2
Mohamed, 2013 (101)	n.r.	Mixed	Eastern Mediterranean	Egypt	500	n.r.	41.0
Moller, 2008 (65)	2005	Male	Europe	Kyrgyzstan	1004	1.0	32.6
Monsalve-Castillo, 2009 (91)	n.r.	Male	Americas	Venezuela	200	n.r.	31.6
Naik, 2014 (37)	n.r.	Male	South-East Asia	India	1600	0.0	n.r.
Narkauskaite, 2007 (35)	2003	Male	Europe	Lithuania	1122	86.5	27.8
Narkauskaite, 2007 (35)	2003	Female	Europe	Lithuania	67	86.5	34.4
Narkauskaite, 2010 (38)	2009	Female	Europe	Lithuania	71	72.2	34.0
Navadeh, 2013 (68)	2009	Male	Eastern Mediterranean	Iran	4536	17.9	n.r.
Nevárez-Sida, 2012 (53)	2006	Mixed	Americas	Mexico	1223	10.0	n.r.
Niriella, 2015 (103)	n.r.	Male	South-East Asia	Sri Lanka	325	0.8	42.0
Niriella, 2015 (103)	n.r.	Female	South-East Asia	Sri Lanka	68	0.8	42.0
Nokhodian, 2012 (94)	2009	Female	Eastern Mediterranean	Iran	163	0.0	34.5
Olubodun, 1996 (80)	n.r.	Male	Africa	Nigeria	81	3.6	27.7
Puga, 2017 (48)	2014	Male	Americas	Brazil	2848	10.7	31.6
Puga, 2017 (48)	2014	Female	Americas	Brazil	520	10.7	31.1
Quitete, 2012 (31)	n.r.	Female	Americas	Brazil	134	3.6	31.5
Ravlija, 2014 (52)	2011	Male	Europe	Bosnia and Herzegovina	620	n.r.	34.0
Reed, 2009 (56)	n.r.	Female	Americas	Brazil	377	27.3	n.r.
Romanian Monitoring Centre for Drugs and Drug Addiction (RMCDD), 2007 (49)	2010	Male	Europe	Romania	2064	n.r.	n.r.

Romanian Monitoring Centre for Drugs and Drug Addiction (RMCDD), 2011 (60)	2006	Male	Europe	Romania	3218	n.r.	n.r.
Santos, 2011 (72)	2010	Mixed	Americas	Brazil	422	18.7	32.7
Santos Barros, 2013 (90)	2008	Female	Americas	Brazil	148	1.3	n.r.
Sawitri, 2016 (70)	2009	Male	South-East Asia	Indonesia	200	n.r.	32.8
Sawitri, 2016 (70)	2009	Female	South-East Asia	Indonesia	300	n.r.	32.8
Sieminska, 2006 (42)	n.r.	Male	Europe	Poland	907	0.0	32.3
Sieroslawski, 2001 (62)	2001	Male	Europe	Poland	1189	n.r.	n.r.
Sieroslawski, 2007 (63)	2007	Male	Europe	Poland	1240	11.9	n.r.
Silva, 2014 (86)	2013	Female	Americas	Brazil	33	26.6	n.r.
Sniķere, 2003 (64)	2003	Mixed	Europe	Latvia	2867	38.8	n.r.
Sniķere, 2010 (51)	2010	Mixed	Europe	Latvia	1965	56.9	31.7
Strazza, 2004 (99)	2000	Female	Americas	Brazil	290	3.0	31
Tiwari, 2014 (25)	n.r.	Mixed	South-East Asia	India	506	41.5	35.8
Turan, 2015 (36)	2013	Male	Europe	Turkey	109	13.5	37.2
United Nations Office on Drugs and Crime (UNODC), 2009 (74)	2008	Mixed	Africa	Uganda	459	8.2	n.r.
United Nations Office on Drugs and Crime (UNODC), 2010 (58)	n.r.	Male	Eastern Mediterranean	Afghanistan	92	n.r.	n.r.
Vaz, 1995 (66)	1991	Male	Africa	Mozambique	1284	43.6	26.2
Vaz, 1995 (66)	1991	Female	Africa	Mozambique	54	27.0	29.2
Weilandt, 2007 (50)	n.r.	Male	Europe	Armenia	542	2.5	33.7
Winetsky, 2014 (26)	2010	Male	Europe	Tajikistan	1317	2.4	36.0
Zamani, 2010 (59)	2007	Male	Eastern Mediterranean	Iran	203	n.r.	34.8
Zhang, 2010 (84)	2006	Male	Western Pacific	China	313	2.0	30.4
Zhang, 2010 (84)	2006	Female	Western Pacific	China	201	2.0	30.5
Zhang, 2012 (106)	2011	Male	Western Pacific	China	12380	n.r.	n.r.
Zhu, 2017 (98)	2013	Female	Western Pacific	China	2709	7.1	n.r.

Ziaee, 2014 (95)	2010	Mixed	Eastern Mediterranean	Iran	881	2.1	34.7

Web Table 11. Pooled **fixed-effects** prevalence estimates of substance use before and during imprisonment in low- and middle-income countries. CI, confidence interval.

Substance	Before imprisonment			During imprisonment		
	Prevalence (%)	95% CI		Prevalence (%)	95% CI	
		Lower	Upper		Lower	Upper
Nicotine use	70.1	69.5	70.8	56.7	56.2	57.6
Alcohol use	69.5	68.8	70.1	14.0	13.5	14.4
Illicit drug use	40.5	40.0	40.9	23.1	22.6	23.6
Cannabis use	31.0	30.5	31.6	20.5	20.0	21.0
Cocaine use	20.9	20.4	21.4	6.2	5.9	6.5
Opiate use	10.6	10.3	10.9	6.4	6.1	6.7
Injection drug use	7.4	7.2	7.6	1.9	1.7	2.0
